# Co-occurrence of transcriptionally distinct persister cell states underpins neoadjuvant therapy resistance in triple‑negative breast cancer

**DOI:** 10.1186/s13073-026-01643-9

**Published:** 2026-04-09

**Authors:** Yu Zhang, Fatemeh Ahmadi Moughari, Ioanna Mavrommati, Nora J. Doleschall, Kate Moore, Gareth Muirhead, Ram Rajaram Srinivasan, Ping Gong, Jonathan T. Lei, Amy Fleming, Hwei Minn Khoo, Naomi Guppy, Gabrielle Elshtein, Mohammed Inayatullah, Vijay K. Tiwari, Lacey E. Dobrolecki, Michael T. Lewis, Syed Haider, Rachael Natrajan

**Affiliations:** 1 Breast Cancer Now Toby Robins Research Centre, The Institute of Cancer Research, London, UK; 2 Lester and Sue Smith Breast Center, Baylor College of Medicine, Houston, TX United States; 3 Dan L Duncan Cancer Center, Baylor College of Medicine, Houston, TX United States; 4 Institute for Molecular Medicine, University of Southern Denmark, Odense, Denmark; 5 Wellcome-Wolfson Institute for Experimental Medicine, School of Medicine, Dentistry & Biomedical Science, Queens University Belfast, Belfast, UK

**Keywords:** Triple-negative breast cancer, Residual disease, Neoadjuvant chemotherapy resistance, Drug-tolerant persister cells, KDM5B, Hypoxia, Single-cell transcriptomics

## Abstract

**Background:**

Although the addition of neoadjuvant immune checkpoint blockade to chemotherapy has improved patient outcome in early triple‑negative breast cancer (TNBC), some patients continue to have poor disease outcomes.

**Methods:**

To characterise neoadjuvant chemotherapy (NAC) resistant cell populations that persist post-NAC, we created a high-resolution single-cell atlas of 129,433 cells from fourteen TNBC patient-derived xenograft (PDX) models with residual disease post-NAC. We identified transcriptionally distinct cancer subpopulations or cell states using unsupervised clustering and characterised‑ their regulatory network as well as clinical associations with treatment response, metastatic progression and survival. Findings were validated using multiple independent TNBC cohorts.

**Results:**

Four major transcriptionally distinct cancer cell states were identified that were shared across the PDX models. These cell states were characterised by enrichment of developmental, hypoxia, interferon signalling, chromosomal instability, and DNA-damage pathways. Hypoxia and interferon signalling cell states shared transcriptional features of drug-tolerant persister cells. Hypoxia cell states contained both cycling and non-cycling cells and were not associated with mutation status. Extensive validation in TNBC patient cohorts confirmed that dysregulated pathways enriched in residual disease were pre-existing in chemotherapy-naïve patients, maintained in distant metastases and were both prognostic and predictive of treatment response. *In vitro* functional validation highlighted that inhibition of the lysine demethylase KDM5B suppressed the emergence of drug‑tolerant persister cells.

**Conclusions:**

Neoadjuvant therapy selects for epigenetically regulated, hypoxia-related cycling and non-cycling drug‑tolerant persister cells in TNBC. These cell states are maintained in metastatic disease and are prognostic/predictive of therapy response.

**Supplementary Information:**

The online version contains supplementary material available at 10.1186/s13073-026-01643-9.

## Background

Triple‑negative breast cancer (TNBC) is an aggressive disease that lacks expression of the oestrogen and progesterone hormone receptors, and lacks amplification and over-expression of the receptor tyrosine-protein kinase *ERBB2* (HER2). While immunotherapy is now a standard addition in the neoadjuvant systemic therapy regimen, almost half of patients with TNBC show a high residual cancer burden (RCB) following chemotherapy, which is linked with poor metastasis-free and overall survival rates [1, 2]. It is therefore crucial to elucidate mechanisms and biomarkers predictive of therapy resistance to improve treatment modalities for TNBC patients with a high RCB post neoadjuvant therapy.

Emerging evidence suggests that non-genetic mechanisms play a key role in driving chemoresistance, rather than selection for recurrent genetic mutations or copy number alterations (CNA) in residual disease [3]. Recent studies in TNBC have shown that subclonal minority populations of transient “drug-tolerant persister” (DTP) epithelial cells, cells that survive initial therapy with reversible and heterogeneous phenotypes, mediate resistance through reversible epigenetic mechanisms under the selective pressure of therapy [4–7]. Moreover, “transcriptionally primed” cells, which share some features of those seen in residual disease, have been documented at subclonal levels in treatment-naïve disease and are enriched in residual disease [8]. Notably, a recent study identified pre-existing drug‑tolerant persister cells in TNBC, characterised by the presence of a subclonal population of cells harbouring a bivalent chromatin state, that were marked by both histone H3 lysine‑27 trimethylation (H3K27me3) repressing and histone H3 lysine‑4 trimethylation (H3K4me3) activating epigenetic marks. This suggests that pre-existing minority populations of cells have the capability to quickly activate gene expression programmes under the selective pressure of chemotherapy to enable them to survive [7]. Single-cell studies of treatment-naïve TNBC have also highlighted the pre-existence of treatment resistant minority subpopulations of cells that are enriched for pathways associated with innate immune sensing and glycosphingolipid function which together as a signature associate with poor patient prognosis [9]. Together, these studies suggest that both pre-existing and acquired transcriptional alterations underpin therapy resistance in TNBC.

To understand whether residual cancer cells after neoadjuvant chemotherapy (NAC) harbour stably propagated transcriptional alterations once therapy is removed, we characterised the intra-tumour transcriptomic heterogeneity of fourteen TNBC patient-derived xenograft (PDX) models generated from post-NAC tumours, using high depth single-nuclei sequencing (snRNA-seq). Based on the hypothesis that TNBC tumours with viable residual cancer cells post-NAC harbour enrichment of chemotherapy resistant cell states that remain stably selected for post chemotherapy exposure, we aimed to (i) identify and characterise potential chemoresistance associated cell states and (ii) provide a valuable resource for functional interrogation of the subclonal heterogeneity of NAC resistant disease.

## Methods

### *In vivo* PDX growth

Fourteen TNBC PDX models were generated as previously described [10–13]. Five PDX models established and grown at Baylor College of Medicine (BCM), three at the Huntsman Cancer Institute (HCI), and six at the Jackson Laboratory (JAX). Detailed information regarding sample origin and clinical metadata is provided in Additional file 1: Table S1 [14, 15]. All BCM mouse studies were conducted in accordance with the *NIH Guide for the Care and Use of Laboratory Animals* and approval by the BCM Institutional Animal Care and Use Committee (IACUC). Procedures involving HCI animals were approved by the University of Utah IACUC, and all JAX animal procedures were performed under protocols approved by the Jackson Laboratory IACUC.

Tissue fragments from TNBC PDX models ranging from passage one to passage nine were transplanted into the cleared inguinal #4 mammary fat-pad of three- to four-week-old SCID/beige mice (Inotiv; C.B-17/IcrHsd-PrkdcscidLystbg-J) for BCM models [13] or NOD.Cg-Prkdc^scid^ Il2rg^tm1Wjl^/Sz mice for HCI [11] and JAX models. Tumours were harvested before reaching a maximum volume of 1500 mm^3 and were flash frozen in liquid nitrogen. Complete growth kinetics for individual mouse of the BCM and HCI models treated with carboplatin and docetaxel are provided in our companion study [16]. All PDXs were routinely tested for pathogens using the IDEXX screening panel and were authenticated by short tandem repeat (STR) profiling every five transplant generations. These PDX models were subjected to whole exome sequencing (WES), snRNA-seq, Immunofluorescence (IF), and Immunohistochemistry (IHC).

### WES data analysis 

We performed WES on seven PDX models: J000080739, J000103634, J000108157, TM00091, TM00107, TM01278, and HCI-037. BBSplit from the BBMap package (BBMap – Bushnell B., RRID: SCR_016965) was used to map the raw sequencing reads to human GRCh38 reference genome [17] and mouse GRCm38 reference genome (GENCODE, release M15), simultaneously. Reads mapping to the human reference genome were retained for downstream analysis.

Paired-end FASTQ reads were quality checked using FastQC (RRID: SCR_014583), and trimmed using Trim Galore [17, 18] v0.6.6 (RRID: SCR_011847). Reads were then aligned to the human GRCh38 reference genome [17] using Burrows-Wheeler Aligner [19] v0.7.17 (RRID: SCR_010910) with default parameters. Duplicate reads were removed by Picard Tools [20] v2.23.8 (RRID: SCR_006525). Variant calling was followed the Genome Analysis Toolkit (GATK) Best Practices [21, 22], using GATK [22] v4.1.9.0 (RRID: SCR_001876) and MuTect2 [23]. The average depth of coverage achieved by WES across all samples was 59× (range: 33.93–80.83×). We used a generic panel of normals for hg38 from GATK [24] to serve as the normal reference in MuTect2 variant caller. SnpEff [25] (RRID: SCR_005191) and SnpSift [25, 26] v5.1 (RRID: SCR_015624) were used to annotate and predict the effects of genetic variants using GRCh38.105 reference, dbNSFP v4.4a database, ClinVar [27] release 20230,30, and gnomAD exome [28] v2.1.1. CNA analysis was performed using CNVkit [29] v0.9.9 (RRID: SCR_021917). Variants and CNA of BCM-0046, BCM-0104, BCM-4849, BCM-5471, BCM-7821, HCI-001, and HCI-027 were downloaded from the Baylor PDX portal [10, 30].

Variants that satisfy all of the following criteria were retained for downstream analyses: (i) splice-site variants or exonic variants with known functional consequences, excluding synonymous single nucleotide variants (SNVs), (ii) read depth larger than or equal to 5, (iii) HIGH or MODERATE impact as defined by SnpEff, and (iv) variants which were either not found in gnomAD or have gnomAD non-cancer allele frequency < 0.001 or with an annotation in ClinVar. For CNA analysis, standard deviation (SD) of log_2_ fold change (FC) ratio of CNA was calculated for each sample. Log_2_(FC) ratios was then standardised by dividing by the sample-specific SD. Standardised values > 3 were considered as copy number gains, whereas values < -3 were considered to be copy number losses.

### Single-nuclei RNA library preparation and sequencing

We performed snRNA-seq on fourteen TNBC PDX models. Frozen tissue was weighed and cut into small pieces using a chilled razor blade on dry ice and 25-40 mg of tissue transferred into a 7 ml douncer (Millipore Sigma, #D9063) on ice for single nuclei preparation. One millilitre of cold lysis buffer comprising 0.32 M Sucrose, 5 mM CaCl_2_, 3 mM Mg(Ac)_2_, 20 mM Tris-HCl pH 7.5, 0.10% Triton X-100, 0.1 mM EDTA pH 8.0, DEPC Water, 40 U/mL RNase Inhibitor (Millipore Sigma, #3335402001) was added to the douncer, and the tissue was incubated for a 5-minute lysis. The tissue was homogenised with 2-5 strokes with the loose pestle (pestle A) and 2-5 strokes with the tight pestle (pestle B). After the 5-minute lysis, nuclei were pelleted by spinning at 4°C for 10 min at 800 x g. The supernatant was removed and the nuclei pellet was resuspended with 2 mL wash buffer comprising 1x PBS, 1% BSA, and 0.1 U/µL RNase Inhibitor. Centrifugation and resuspension with wash buffer were repeated for a total of two washes. After the second wash, the nuclei suspension was filtered using a 40 µm Flowmi cell strainer (Millipore Sigma, #BAH136800040). The nuclei suspension was stained with DAPI for fluorescence‑activated cell sorting (FACS) sorting. DAPI-positive nuclei were collected, while debris and nuclei aggregates within the DAPI-positive gating were excluded. After FACS, sorted nuclei were spun down at 4°C for 10 min at 800 x g. Nuclei counting was performed using a haemocytometer (Thermo Fisher, #22-600-100). Following counting, the appropriate volume for each sample was calculated for a target capture of 10,000 nuclei and loaded onto the 10x Genomics Single-Cell 3′ v3.1 platform (G chip). After droplet generation, samples were transferred to pre-chilled PCR strip tubes (MJS BioLynx, #US14024700) and incubated overnight in a Veriti 96-well thermal cycler (Thermo Fisher). The next day, cDNA was recovered using 10x Genomics Recovery Agent and subsequently cleaned up using a Silane DynaBead mix as outlined by the user guide 3′ Reagent Kits v3.1. Purified cDNA was amplified for 11 PCR cycles before being cleaned up using SPRIselect beads (Beckman Coulter, #B23318). Samples were run on a Bioanalyzer (Agilent Technologies) to determine cDNA concentration. Libraries were prepared as outlined by the Single-Cell 3′ Reagent Kits v3.1 user guide with modifications to the PCR cycles based on the calculated cDNA concentration. The molarity of each library was calculated based on library size as measured using a Bioanalyzer (Agilent Technologies) and qPCR amplification data (Roche, #07960140001). Samples were sequenced on Illumina’s NovaSeq 6000 with the following run parameters: Read 1, 28 cycles; Read 2, 90 cycles; Index 1, 10 cycles; Index 2, 10 cycles.

### Cell species identification

CellRanger [31] v6.0.2 (10x Genomics; RRID: SCR_017344) was used to align reads from the FASTQ to the GRCh38 and mm10 transcriptome reference (Cell Ranger reference version 2020-A) and generate feature-barcode matrices. Downstream analyses were performed using Seurat v4.0.3 (RRID: SCR_016341). For each sample, features detected in at least 5 cells were kept.

The identification of cell species was based on the CellRanger multi-genome multiplets detection method [32], which identifies multiplets based on differential alignments to human and mouse genomes. For each sample, thresholds distinguishing human and mouse nuclei were defined as the 10th percentile of all nuclei in which the unique molecular identifier (UMI) counts for one species exceeds those of the other. We annotated the human nuclei as those whose human UMI counts exceeding the human threshold, mouse UMI counts below the mouse threshold, and a higher number of human counts than mouse UMI. Nuclei not meeting these criteria were excluded from further analysis.

### Quality control

Filters were applied to all human cells within each individual sample to only include true cells with high quality. The lower and upper thresholds for the number of reads and UMI counts per cell were determined by identifying the local minima in density distributions (Additional file 1: Table S2). Cells with < 5% of transcripts mapping to mitochondrial genes were kept. Doublet detection was performed using DoubletFinder [33] v2.0.3 (RRID: SCR_018771) with 50 principal components (PCs), *pk* = 0.09, and *pN* = 0.25, and scDblFinder [33, 34] v1.6.0 (Bioconductor; RRID: SCR_022700) with default parameters. If the number of doublet detected by both methods exceeds the estimated multiplet rate reported by 10x Genomics [35] (*ndoublet*), the top *ndoublet* cells ranked by DoubletFinder scores were removed; otherwise, all intersecting doublet cells were removed. A summary of raw and filtered data for each sample can be found in Additional file 1: Table S2.

### Normalisation and dimensionality reduction

SCtransform normalisation enables recovering sharper biological distinction compared to log-normalisation [36]. Normalisation and variance stabilisation were performed using SCtransform [37] v2 (RRID: SCR_022146) with 3000 variable features, regressing out the percentage of mitochondrial reads per cell, percentage of ribosomal protein large subunit (RPL) per cell, number of genes per cell, and number of UMI per cell. Cell cycle phases were determined by “CellCycleScoring” function in Seurat using the built-in G_2_M and S phase gene sets to separate cells in G_1_ and S/G_2_M phases. Principal component analysis (PCA) was performed with initial 100 PCs. The optimal number of PCs was determined by the elbow point, defined as the first PC after which the percent change in variation between consecutive PCs was less than 0.1%. The optimal number of PCs was then used to construct the nearest-neighbour graph using “FindNeighbors” function and to compute Uniform Manifold Approximation and Projection (UMAP) using “RunUMAP” function in Seurat v4.0.3.

### Within sample clustering

We clustered cells within each sample separately for the G_1_ phases and S/G_2_M phase to avoid the confounding effects introduced by the cell cycle transcriptional programmes. Davies–Bouldin index [38] was used to determine the optimal resolution to identify the clusters by searching resolutions ranging from 0.01 to 0.1 with gap 0.01 and 0.2 to 1 with gap 0.05, provided that the resulting number of clusters ranged from 3 to 8 (Additional file 1: Table S2). To ensure methodological consistency, the similarity matrix obtained from “FindNeighbors” function in Seurat was scaled to the interval [0, 1] and used as the distance matrix in Davies–Bouldin index calculations. The clusters obtained within individual samples were defined as initial clusters.

### Meta clustering

We used CIDER [39] meta clustering framework to obtain the clusters across samples. CIDER calculates the inter-group similarities between every pair of initial clusters from different samples and generates an inter-cluster similarity matrix of all initial clusters from all samples for clustering. Instead of using CIDER’s default input of all gene expression values, we provided the log-normalised SCtransform data restricted to selected highly variable genes (HVGs) and used Spearman's correlation to calculate pairwise distance coefficients. The selected HVGs were defined as genes identified as variable in individual samples and shared by at least three samples across all fourteen PDX models (both G_1_ and S/G_2_M). We then applied a hierarchical clustering on the inter-cluster similarity matrix and used a cutoff of 0.75 to define meta clusters (MCs).

### Differential gene expression analysis

Differential gene expression analysis was performed within individual samples first and subsequently combined to determine the marker genes for MCs. For each sample, initial clusters belonging to the same MC within a sample formed a subcluster. The markers of each subcluster were defined as the genes that were significantly over-expressed in that subcluster compared to all other subclusters within that sample, identified using “FindAllMarkers” Seurat function with parameters: *return.thresh* = 1, *logfc.th* = 0, and restricting features to the HVG set used for meta clustering; all other parameters were left as default values from Seurat v4.0.3. After identifying subcluster markers on each sample, markers of all subclusters in each MC, excluding heat-shock protein genes, mitochondrial genes, and RPL genes were aggregated using Fisher’s combined probability test as follows:

$$\:{{\upchi\:}}_{2k}^{2}=\:-2\sum_i^kln{p}_{i}$$

where $\:{{\upchi\:}}_{2k}^{2}$ follows a chi-squared distribution with $\:2k$ degrees of freedom, $\:{p}_{i}$ is the *P* value from sample $\:i$, and $\:k$ denotes the total number of samples contributing to the MC. Combined *P* values were adjusted for false discovery rate (FDR) using Benjamini–Hochberg correction. MC marker genes were defined as genes that were significantly over-expressed in the cells from that MC compared to all other cells and were defined as those with FDR-adjusted *P* < 0.01 and at least 2 samples showing log_2_(FC) > log_2_(1.25). Marker genes were ranked by the combined *P*. Top 100 marker genes were used for further analyses.

### Gene sets similarity analysis

The overlap coefficient (OC) was used to evaluate the similarities between two gene sets. OC is calculated by dividing the size of the intersection of two sets by the size of the smaller set:

$$\:OC\left(A, B\right)=\frac{|A\ \cap \:B|}{\mathrm{m}\mathrm{i}\mathrm{n}(\left|A\right|,\:|B\left|\right)}$$

where $\:\left|A\right|$ is the size of set $\:A$.

### Pathway and gene set enrichment analysis

The Fisher’s exact test was performed on the hypergeometric distribution (function “phyper” from stats v4.4.1.0 (RRID: SCR_025968). Top 100 marker genes from each MC were tested on the Reactome pathway database (RRID: SCR_003485) from MSigDB [40] v7.5.1, restricting the analysis to gene sets containing 3–500 genes. Three additional cancer-related signatures, including AKT1 signalling via mTOR, angiogenesis, and hypoxia were included as described by a previously published study [8].

Let *N* represent the size of genes in the filtered merged Seurat object, *K* the size of each pathway gene set, *n* the size of marker genes (here *n* = 100), and *k* the size of the intersection between marker genes and pathway gene set. The *P* value can be calculated as the cumulative probability:

$$\:P\left(X\ge\:k\:\right|n, N, K)=\sum\:_{i=k}^{n}\frac{\left(\genfrac{}{}{0pt}{}{K}{i}\right)\left(\genfrac{}{}{0pt}{}{N-K}{n-i}\right)}{\left(\genfrac{}{}{0pt}{}{N}{n}\right)}$$

Pathways intersecting marker genes at ≥ 3 genes and Benjamin–Hochberg FDR-adjusted *P* < 0.05 were considered significant.

Gene set variation analysis (GSVA) was performed using the “gsva” function from GSVA [41] v1.42.0 on the gene expression matrices.

### Transcription regulatory network inference

Single-cell regulatory network inference and clustering (SCENIC) [42] v1.3.1 is a tool to infer gene regulatory networks (GRN) and predict regulon activities from scRNA-seq data. The GRN was built using GRNBoost embedded in SECNIC, followed by identification of transcription factor (TF)–target regulons using motif enrichment against the cisTarget DNA‑motif database [43]. AUC-based regulon activity scoring (AUCell) was used to comput regulon activity scores within individual nuclei.

### Identification of MC-specific TFs

For each sample, we performed Wilcoxon rank-sum test on the AUCell scores for each TF obtained from SCENIC between cells from the target MC and other MCs. Significant TFs were selected as (i) *P* < 0.05, (ii) mean AUCell difference between the target MC and other MCs > 0.1 within the sample, and (iii) regulon or its extended gene sets detected in at least two samples. MC-specific regulons were defined as the ones with higher median AUCell scores in target MC than other MCs and only specific to one MC.

Statistical significance of the OC between TF-regulated gene sets and the top 100 MC marker genes was assessed using a *t*-test on OC values, with *P* < 0.05 considered significant.

### Gene signatures and calculation

We applied four signatures identified from scRNA-seq data including persister signature [7], metastatic burden signature (MBS) [44], epithelial-mesenchymal plasticity (EMP) signature [45], and micrometastasis signature [46], and five signatures identified from microarray and/or bulk RNA-seq data including residual tumour signature (RTS) [47], prognostic signature (PS) [48], chromosomal instability 70 gene signature (CIN70) [49], p53 mutant vs. wild-type [50], and recombination proficiency score (RPS) [51] to our data. Methods for computing gene set scores are described below:

### Persister signature

The persister signature consists of genes identified exclusively in a group of cells that survived initial chemotherapy in TNBC [7]. GSVA scores were calculated for the persister gene set.

### Metastatic burden signature (MBS)

MBS is a gene signature upregulated in metastatic cells from tissues with high metastatic burden [44]. GSVA scores were calculated on MBS.

### Epithelial-mesenchymal plasticity (EMP) signature

EMP signature reflects transcriptional plasticity, enabling dynamic switching between epithelial and mesenchymal cell states [45]. Twenty-six overlapping genes identified from different single-cell technologies in the original study [45] were used to define the EMP signature. GSVA scores were calculated on EMP signature.

### Micrometastasis signature

The micrometastasis signature is a set of genes upregulated in micrometastasis cells [46]. GSVA scores were calculated on this signature.

### Residual tumour signature (RTS)

RTS genes were identified by molecular profiling of residual tumours after NAC, which may be associated with drug resistance [47]. For each MC within each sample, pseudo-bulk expression was calculated as the mean log-normalised expression per gene. RTS scores were calculated as the mean of pseudo-bulk expression of all RTS genes.

### Prognostic signature (PS)

PS is composed of genes related to cell cycle, invasion, metastasis, and angiogenesis, identified from DNA microarray profiling of 117 breast cancer (BC) patients without tumour cells in local lymph nodes at diagnosis [48]. Pseudo-bulk expression was calculated per MC and sample, and PS scores were calculated as mean of pseudo-bulk expression of all genes in PS.

### Chromosomal instability signature

CIN70 is a signature of chromosomal instability from specific genes whose expression consistently correlates with total functional aneuploidy in multiple cancer types [49]. Pseudo-bulk expression was calculated per MC and sample, and CIN70 scores were then calculated as the mean of pseudo-bulk expression value of all genes in CIN70.

### p53 mutant vs. wild-type signature

This signature was derived by the differential gene expression analysis on *TP53*-mutant and *TP53*-wild-type tumours and cell lines [50]. Pseudo-bulk expression was calculated per MC and sample, and the first PC of pseudo-bulk expression of the gene set was used as p53 signature score.

### Recombination proficiency score (RPS)

RPS quantifies the efficiency of DNA repair pathways in the context of cancer therapy based on the expression levels of four genes, including *RIF1*,* PARI*,* RAD51*, and *XRCC5* (*Ku80*). High expression of these genes are associated with a low RPS [51]. Pseudo-bulk expression was calculated per MC and sample as the mean of scaled log-normalised gene expression (“scale.data” slot from RNA assay in Seurat object). The RPS score was then calculated as the sum of pseudo-bulk expression of all genes in RPS multiplied by -1.

### Correlation of metastatic signatures

We computed cell-wise similarity between the MBS and micrometastasis GSVA scores using cosine similarity. For each cell $\:i$, let $\:{x}_{i}$ and $\:{y}_{i}$ denote the GSVA scores of MBS and micrometastasis signatures, respectively. The similarity score for cell $\:i$ was calculated as:

$$\:{r}_{i}=\:\frac{{x}_{i}\:.\:\:{y}_{i}}{\sqrt{{x}_{i}^{2}+{y}_{i}^{2}}}$$

We scaled the similarity scores to the interval [0, 1] for visualisation to highlight the differences and shared patterns across cells.

### Immunofluorescence and immunohistochemistry of marker proteins

Formalin‑fixed paraffin‑embedded (FFPE) PDX tissues were sectioned on a microtome to 5 μm thickness, fixed onto glass slides and dried. Four sections were then used, one for Haematoxylin and Eosin staining, one for CAIX IHC, and two for IF. IHC was carried out using the Dako/Agilent Autostainer Link 48 with PT module retrieval (Dako TRS, pH 6). CAIX antibody (Abcam, ab15086) was applied at 1:1000 and detected using the Rabbit ImmPRESS polymer-HRP (Vector Laboratories). IF staining was performed according to standard protocols. Briefly, tissue sections were deparaffinised in xylene for 15 min, then rehydrated by incubation in decreasing concentrations of ethanol for 2 min each, followed by rinsing in water. Antigen retrieval was performed using citric acid based unmasking solution (Vectorlabs, H-3300, pH 6), at 95°C for 20 min. Slides were cooled in unmasking solution and rinsed in water. Sections were outlined using hydrophobic PAP pen followed by blocking in blocking buffer (Cell Signaling Technology, #12411) for 45 min at room temperature. Antigens were tagged overnight at 4°C using the following primary antibodies: anti-NDRG1 (Thermo Fisher, MA5-32730, 1:500), anti-VCAM1 (Abcam, ab134047, 1:200) and anti-Ki67 (Agilent, #M724001-2, 1:500). Slides were washed in PBS with 0.1% Tween-20 three times for 15 min each. Samples were incubated with Alexa Fluor 488 conjugated anti-rabbit (Invitrogen, A27034) and Alexa Fluor 555 conjugated anti-mouse (Invitrogen, A21422) secondary antibodies at 1:400 dilution for 1 h at room temperature in the dark. Both primary and secondary antibodies were diluted using antibody diluent (Epredia, #12643957). Slides were washed again with PBS with Tween-20 three times for 15 min each and then incubated with 50 ng/ml DAPI in PBS for 15 min at room temperature in the dark. Following an additional 15-minute PBS and Tween-20 wash, coverslips were mounted onto the slides using anti-fade mounting medium (Enzo Life Sciences, ENZ-53002).

Histology and IHC slides were imaged on the Hamamatsu Nanozoomer XR in bright-field at 20X magnification, while representative images were acquired from the IF labelled slides using super-resolution spinning-disk confocal microscopy. Bright-field images were exported using NDP.view v2.9.29. Fluorescent confocal images were processed in ImageJ v1.54p (RRID: SCR_003070). Processing included Z-projection of maximum intensities of multiple focal planes, adjustment of LUTs, modification of channel colours and addition of 50 μm scale bars. IHC slides were quantified using QuPath [52] v0.5.1 (RRID: SCR_018257) using positive cell detection algorithm. H-scores were calculated based on three intensity thresholds to detect different levels of staining in the tumour cell compartment.

### Cell culture and conditions

All cell lines except 4T1 cells were obtained from the American Type Culture Collection (ATCC). The 4T1 cell line was provided by Professor Clare Isacke (The Institute of Cancer Research, London). All cell lines were grown in complete DMEM (Gibco) supplemented with 10% FBS, except for BT-20 cells, which were cultured in α‑MEM (Gibco) with 20% FBS. All cells were grown at 37°C in a humidified incubator with 5% CO_2_.

### 2D cell‑based drug sensitivity assays

We assessed drug sensitivity using established TNBC cell lines, including MDA-MB-231, BT-20, HCC1806, MDA-MB-468, and 4T1. For 2D drug sensitivity assays, 1000-2000 cells were seeded per well in a 96-well tissue culture plate overnight (16 h). The next day, cells were exposed to increasing concentrations of cisplatin (Cambridge Bioscience) or paclitaxel (Cayman Chemical), or respective vehicle controls (PBS + 0.1% NaCl or DMSO). Cell viability was assessed after 72 h using CellTitre-Glo (Promega) according to the manufacturer’s instructions.

### KDM5B siRNA knockdown assays

To evaluate KDM5B dependency, siRNA knockdown experiments were performed in both 2D and 3D culture conditions using MDA-MB-231 and 4T1 cells, as previously described [53–55]. siRNA-lipid transfection reagent complexes were generated with 20 nM ON-TARGETplus Non-targeting Control siRNAs control #1 (NT Control), ON-TARGETplus Human or Mouse KDM5B siRNA-SMARTpool or ON-TARGETplus Human or Mouse Ubb siRNA SMARTpool (Horizon Technologies) for MDA-MB-231 (human) and 4T1 (mouse) cells, respectively.

For 2D assays, 500-2000 cells/well were seeded in standard 96-well tissue culture plates (Grenier) overnight and cells were forward transfected the next morning (16 h) using RNAiMax transfection reagent (Themo Fisher). After 72 h, cell viability was assessed using CellTitre-Glo.

For 3D spheroid assays, siRNA-lipid transfection reagent complexes were prepared using Lullaby transfection reagent (OzBiosciences). Complexes were added to an ultra-low attachment 96-well plate (Costar), after which 5000 cells were then seeded per well and plates centrifuged at 1000 × g for 10 min, 4°C. Six replicate wells were seeded per condition. After 7 days, spheroids were subjected to Alamar Blue stain (Thermo Fisher) and visualised at 590 nm on a plate reader. Statistical comparisons were performed using one-way ANOVA with Bonferroni’s multiple test correction.

### Quantitative PCR

Quantitative PCR (qPCR) was performed to confirm knockdown efficiency of KDM5B in MDA-MB-231 and 4T1 cells. Total RNA was extracted using the RNeasy Mini Kit (Qiagen) and was converted to cDNA using qScript Ultra SuperMix (Avantor). qPCR was performed using the Power SYBR green kit (Thermo Fisher) using human *KDM5B* forward primer AGTGGGCTCACATATCAGAGG, reverse primer CAAACACCTTAGGCTGTCTCC , *GAPDH* forward primer GTCTCCTCTGACTTCAACAGCG, and reverse primer sequence ACCACCCTGTTGCTGTAGCCAA. Mouse *Kdm5b* forward primer CTGGGAAGAGTTCGCGGAC, reverse primer CGCGGGGTGAAATGAAGTTTAT, mouse *Gapdh* forward primer CATCACTGCCACCCAGAAGACTG, and reverse primer sequence ATGCCAGTGCCA AGCTTCCCGTTCAG were used for the 4T1 mouse cell line. *KDM5B* (or *Kdm5b*) expression was normalised to *GAPDH* (or *Gapdh*) housekeeping gene and compared to non-targeting (NT) siRNA control. Statistical comparisons were performed using unpaired *t*-tests.

### Drug‑tolerant persister cell assays

Assessment of drug‑tolerant persister cells was performed using MDA-MB-468, BT-20, and HCC1806 TNBC cell lines, seeded at 1 × 10^4^–1 × 10^5^ cells per well in 6-well plates, depending on the growth rate of each cell line. For cisplatin treatment conditions, cell numbers were doubled at seeding. Cells were treated with the cisplatin IC_50_ (Cambridge Bioscience) or vehicle control (PBS + 0.1% NaCl) for each cell line for 3 days. After 3 days, cells were washed with PBS and media were replaced with 30 µM C70 (MedChemExpress) or DMSO vehicle control. After 3 further days, cells were again washed and media were replaced with normal growth media for the duration of the study. Media were replenished as required. Cells were fixed and stained with crystal violet upon appearance of colonies in the Cisplatin + C70 treated wells. Colonies were solubilised in 1 mL per well of 20% acetic acid prior to 100 µL/well being transferred to a 96-well plate in at least triplicate for quantification on a plate reader at 590 nm. Statistical comparisons were performed using unpaired *t*-tests.

### Independent datasets included in this study

In addition to the WES and snRNA-seq generated from our fourteen PDX models, we have analysed several independent cohorts to validate the findings. The details of number of samples, treatments, and accession numbers for each cohort are summarised in Additional file 1: Table S3. Methods for analysing these datasets are described below.

### Pre- and post-NAC TNBC patient cohort

Kim et al. [8] dataset was obtained from SRA: SRP114962 including the processed scRNA-seq data of seven TNBC patients sampled pre- and post-NAC. We calculated the GSVA score using the top 100 marker genes of our MCs in the patients’ data. A paired *t*-test was used to compare the fraction of cells showing corresponding MC characteristics (GSVA > 0) between pre- and post-treatment on each MC. Cells were assigned to the MC with the highest positive GSVA score, or labelled as “unassigned” if all GSVA scores were negative. To investigate differences between sensitive and resistant cells within each MC, we identified the differentially expressed genes between response groups of the cells from the same MC using “FindMarkers” with thresholds log_2_(FC) > log_2_(1.25) and adjusted *P* < 0.05. Pathway enrichment analysis was then conducted using hypergeometric tests on the intersection of these differentially expressed genes and Reactome gene sets. We further applied SCENIC [42] to infer the TF activities as described above on this dataset.

### Metastatic patient cohort

The Klughammer et al. [56] dataset, including single-cell RNA sequencing (scRNA-seq) and snRNA-seq data of 67 metastatic breast cancer patients was downloaded from CELLxGENE website [57]. The malignant cells from TNBC patients were selected and the GSVA scores were calculated using the top 100 marker genes of our MCs.

### Matched primary and metastatic PDX cohort

The Winkler et al. [58] dataset including raw scRNA-seq data from eight matched primary and metastatic basal-like TNBC PDX models were downloaded from the Gene Expression Omnibus (GEO), accession number GSE210283. Data preprocessing followed the standard Seurat workflow, including log-normalisation, scaling, PCA, and UMAP dimension reduction. GSVA scores were subsequently computed using the top 100 marker genes identified for our MCs.

### Treatment-naïve syngeneic 4T1 data

We validated the identified cell states in murine TNBC syngeneic models 4T1 and MMTV-PyMT from Adrover et al. [59]. The raw scRNA-seq data was downloaded from GEO, accession number GSE123366 and were processed using Seurat workflow, including log-normalisation, PCA, and UMAP for dimensionality reduction. GSVA scores were then calculated for *EPCAM*^+^ cells using the top 100 marker genes of our MCs.

### TNBC SCAN-B survival clinical cohort

The Sweden Cancerome Analysis Network–Breast Cohort (SCAN-B) [60] contains bulk RNA-seq data for 231 TNBC patients treated with chemotherapy. The RNA-seq data was downloaded from Mendeley Data [61]. Survival analyses were implemented on both gene set GSVA scores (for top 100 marker genes) and individual gene expression values. Patients were classified into “low” and “high” expression groups based on the median of GSVA score or gene expression. A cox proportional hazards regression model was fit using 10 years survival data, implemented by “coxph” function in survival v3.2-13. An estimate of a survival curve for censored data using the Kaplan–Meier method was computed by “survfit” function in survival v3.2-13, and the Wald test *P* and hazard ratio (HR) were reported with *P* < 0.05 considered as significant.

### Neoadjuvant therapy response clinical cohorts

Three independent cohorts were used for treatment response analyses, including BrighTNess [62], I-SPY2 [63], and Park et al. [64] cohort.

BrighTNess [62] is a phase III randomised clinical trial (NCT02032277) that enrolled 482 TNBC patients treated with NAC. Participants were randomly assigned to three treatment arms in a 2:1:1 ratio. Arm A included 237 patients who received paclitaxel combined with carboplatin and veliparib; Arm B included 122 patients treated with paclitaxel plus carboplatin; and Arm C included 123 patients who received paclitaxel alone. Bulk RNA-seq of BrighTNess dataset was retrieved from GEO under accession number GSE164458.

The I‑SPY2 trial [63] (NCT01042379) is a multi‑arm phase III randomised study that enrolled 987 women with breast cancer, including 363 with triple‑negative disease. Participants were allocated to 13 treatment arms evaluating various investigational neoadjuvant regimens. For our response‑prediction analyses, we focused on TNBC patients from three arms: paclitaxel alone (*n* = 85), paclitaxel plus ABT 888 and carboplatin (*n* = 39), and paclitaxel plus pembrolizumab (*n* = 29). Bulk RNA-seq of I‑SPY2 dataset was downloaded from GEO under accession number GSE194040.

Park et al. [64] cohort which is a dataset of 93 TNBC patient samples pre-, on-, and post-NAC. Bulk RNA-seq data for Park et al. cohort was downloaded from GEO under accession number GSE123845.

In all three cohorts, the patients were labelled as residual disease (RD) or pathological complete response (pCR) according to the treatment response. GSVA scores of top 100 marker genes were calculated on each arm in BrighTNess and I-SPY2. Also, in the Park et al. cohort, GSVA scores of top 100 marker genes were calculated on pre-treatment (T1) and post-treatment (T3) timepoints. To assess GSVA score differences between response groups, one-sided Wilcoxon rank-sum test was performed using “wilcox.test” function from stats v4.11.0. *P* values from seven pre-treatment arms with standard therapy in TNBC were combined using the sum of z method weighted by sample size implemented by “sumz” function from metap v1.5 (RRID: SCR_014686). To evaluate the significance of individual gene between RD and pCR, “lmFit” function from limma v3.50.1 (RRID: SCR_010943) was applied to fit a linear model. The linear model was then used to compute moderated statistics and log-odds of differential expression by empirical Bayes moderation of the standard errors towards a global value using “eBayes” function from limma v3.50.1 (RRID: SCR_010943). The genes with *P* < 0.05 were considered significant.log_2_(FC) was calculated as the ratio of expression between RD and pCR. The regression model between RD and pCR in Park et al. cohort was adjusted by the tumour purity. Additionally, the change in MC GSVA scores between pre-treatment (T1) and post-treatment (T3) in Park et al. cohort was assessed by Wilcoxon rank-sum test.

### KDM5B ChIP-seq dataset

Chromatin immunoprecipitation sequencing (ChIP-seq) datasets for KDM5B in TNBC cell lines—SUM159, MDA-MB-231, and HCC2157—were retrieved from Yamamoto et al. [65] cohort (GEO: GSE46055). Peak files were annotated using BEDTools v2.27.1 (RRID: SCR_006646) by intersecting with the GRCh37 human reference genome. Peaks overlapping gene bodies by at least one base pair were assigned to corresponding gene names. To identify potential functional targets of KDM5B, genes associated with KDM5B-bound regions were intersected with the top 500 marker genes of G_1_-MC1. Statistical significance of the overlap was evaluated using hypergeometric test. Promoter-associated peak intensities were visualised using Gviz [66] v1.38.4, incorporating GeneHancer [67] enhancer region annotations for mammary epithelial and myoepithelial tissues.

### TNBC PDX proteomics dataset

Processed proteomics mass spectrometry data of 46 TNBC PDX models was obtained from Lei et al. [16]. The data contained log_10_-transformed, batch corrected, iBAQ proteomics intensities. The processed proteomics intensities of top MC marker genes were used for visualisation.

### Statistics and reproducibility

All source codes were implemented in R v4.1.0. An FDR adjusted *P* < 0.05 was used as a significance threshold, unless specified particularly. No statistical method was used to pre-determine sample size, and no randomisation was required.

## Results

### Post-NAC TNBC harbour shared transcriptionally distinct cell states

**Fig. 1:**
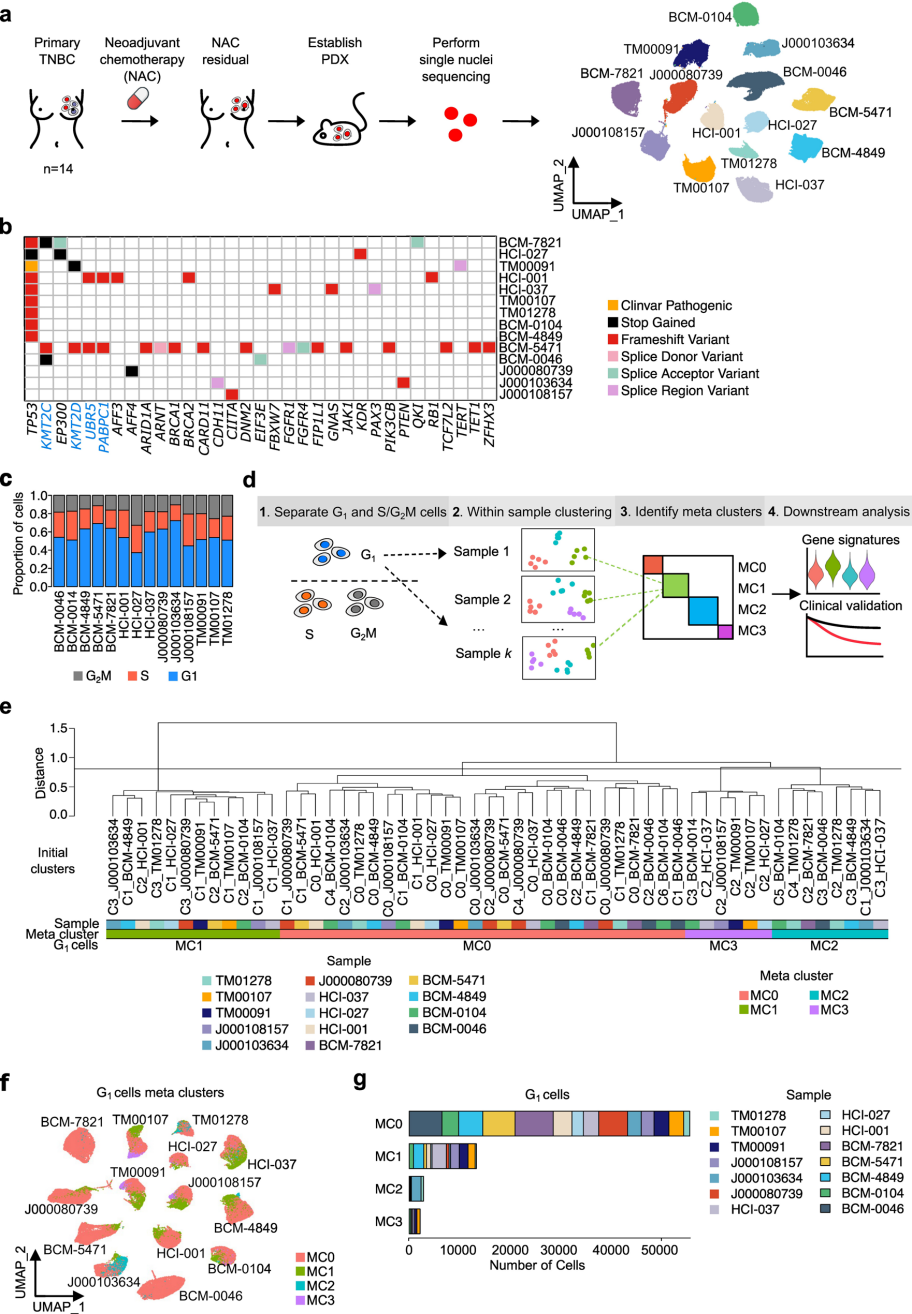
TNBC PDXs derived from residual disease post-NAC share common transcriptional features. **a.** An overview of the atlas and experimental design and UMAP for all cells coloured by sample ID. **b.** Variants of each sample on the COSMIC cancer gene census (CGC, v2018.07.04, https://cancer.sanger.ac.uk/census) tier 1 gene list (gene names in black colour) and recurrent genes from tier 2 gene list (gene names in blue colour). Only the most deleterious variants were shown. In our WES samples, unknown exonic function variants were removed and only “PASS” variants were shown. **c.** Proportion of cells in three cell cycle phases for each sample. **d.** Scheme of the computational workflow. **e.** Dendrogram of meta clustering on G_1_ epithelial cells from the 14 PDX models (C#_indicates the cluster number when the initial clustering was performed on the individual PDXs). **f.** UMAP for G_1_ epithelial cells coloured by MCs. **g.** Number of G_1_ epithelial cells from each sample in each MC

We collected a series of fourteen PDX models established from residual disease after implantation into the mammary fat-pad of female mice from TNBC patients treated with NAC [10, 11] and subjected them to snRNA-seq targeting 10,000 cells and sequencing at an average depth of 150,000 reads per cell (Fig. 1a, Additional file 1: Table S1, and Methods). Additionally, we performed bulk whole exome sequencing (WES) of these models that identified a typical copy-number profile of TNBC including gains of chromosome 1q and 8q (Additional file 2: Fig. S1a). Recurrent deleterious somatic mutations were identified in known breast cancer driver genes [68] *TP53*,* KMT2C*,* EP300*,* KMT2D*,* UBR5*, and *PABPC1*, as well as predicted germline mutations in *BRCA1* and *BRCA2* in two of the models (Methods, Fig. 1b, Additional file 2: Fig. S1b, and Additional file 1: Table S4).

**Fig. 2:**
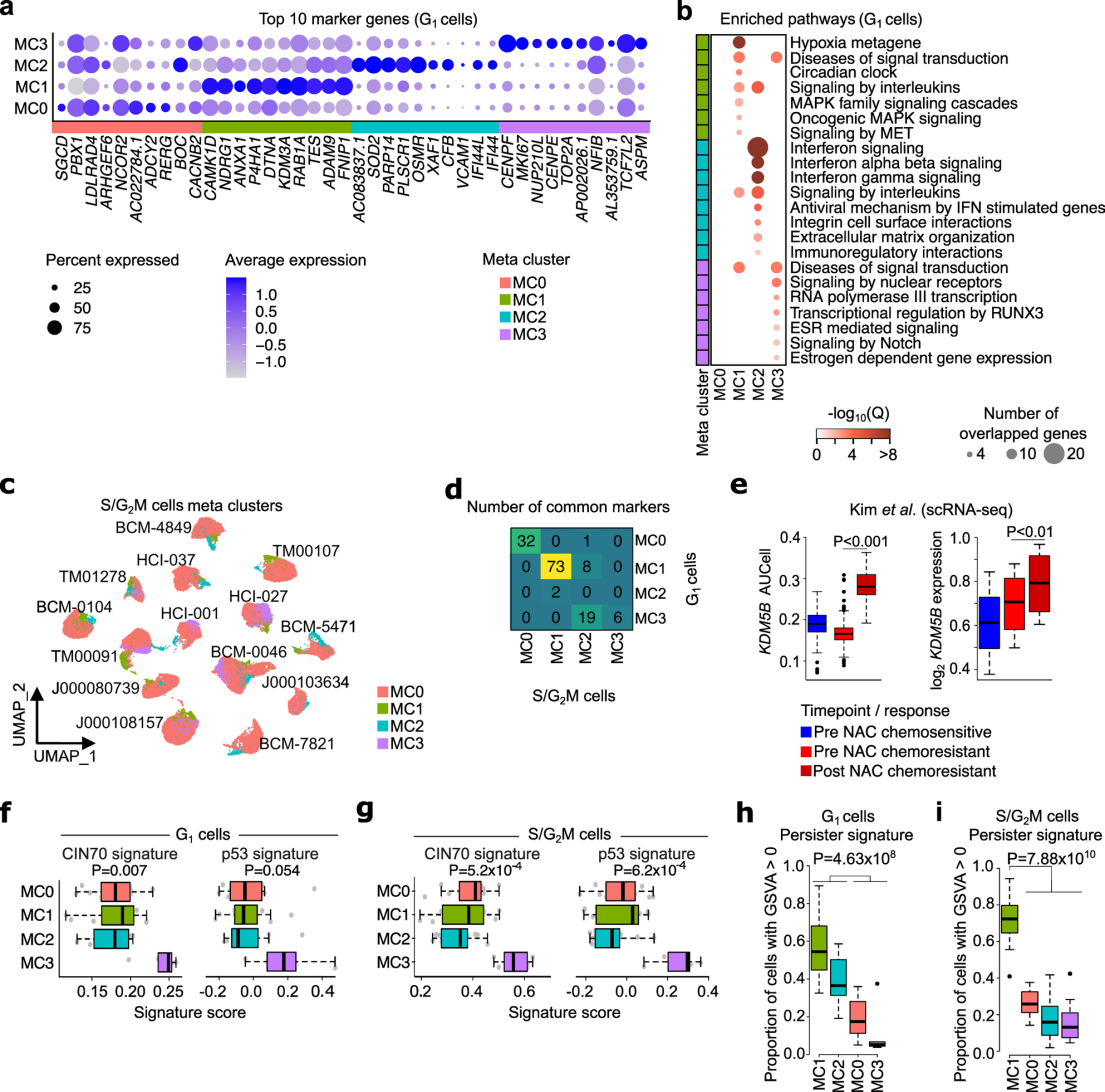
TNBC PDXs derived from residual disease post-NAC harbour transcriptionally defined cell states, showing distinct pathway regulation and signatures. **a**. Dot plot of the top 10 MC marker genes of G_1_ epithelial cells, showing MC specificity. The colour of the dot represents average gene expression, and the size of the dot represents percentage of cells expressing the gene. **b**. Pathway enrichment for all or top 10 most significant pathways in G_1_ epithelial cells from Reactome database in MSigDB40. The colour of dot represents -log_10_(FDR-adjusted *P*) and the size of dot represents the number of genes intersected by the top 100 marker genes and pathway gene sets. **c**. UMAP for S/G_2_M epithelial cells coloured by MCs. **d**. Number of common top 100 marker genes between G_1_ and S/G_2_M MCs. **e**. Box and whisker plots of AUCell score of *KDM5B* TF regulon (left) and *KDM5B* gene expression levels (right) between resistant and sensitive patients pre- and post-NAC in an independent single-cell dataset [8] show significant enrichment of *KDM5B* regulation in resistant patients compared to sensitive patients both pre- and post-chemotherapy (Wilcoxon rank-sum test). **f-g**. Box and whisker plots of signature scores of CIN70 signature and p53 mutant vs wild-type signature on G_1_ (**f**) and S/G_2_M epithelial cells (**g**) show higher scores of MC3 in both G_1_ and S/G_2_M cells (Kruskal-Wallis test). **h**. Box and whisker plots showing the proportion of cells with positive persister signature score (GSVA > 0) on G_1_ cells, highlighting that MC1 and MC2 have significantly higher persister signature-positive cells than other MCs (Wilcoxon rank-sum test). **i**) Box and whisker plots showing the proportion of cells with positive persister signature score (GSVA > 0) on S/G_2_M cells highlighting that MC1 has significantly higher persister signature-positive cells than other MCs (Wilcoxon rank-sum test)

To delineate relative transcriptional changes amongst cancer cells, we focussed on epithelial cells from the single-cell analysis, after removing mouse stromal cells (Methods). In total, 129,433 cells were recovered after quality control (Methods, Additional file 2: Fig. S2a-d). A total of 15,339 genes were found to be common among all PDXs (Additional file 2: Fig. S2e). UMAP clustering of epithelial cells revealed separation of cells according to patient (Fig. 1a). To mitigate the potential loss of biology by co-normalising a large number of snRNA-Seq profiles [69], we used a meta clustering approach (CIDER [39]) to identify shared and distinct epithelial cell populations, hereafter referred to as meta clusters (MCs), amongst samples (Methods). To avoid the confounding impact of cell cycle effects on MCs, and to identify cell states with distinctive transcriptional profiles shared across multiple samples, we initially focussed on the identification of distinct transcriptionally-defined populations using epithelial cells in the G_1_ phase of the cell cycle [70] (73,702 cells, Fig. 1c-d). Clustering of these G_1_ cells revealed four MCs (Fig. 1e-g). We did not observe any association of MCs with any recurrent gene mutations (Fig. 1b and g). Next, we identified the transcriptional marker genes of these MCs, i.e., those that are significantly over-expressed compared to all other MCs with log_2_(FC) > log_2_(1.25) in at least two samples and FDR-adjusted Fisher’s combined *P* < 0.01 (Fig. 2a, Additional file 2: Fig. S3a, and Methods). We found 1,844, 1,257, 774, and 322 significant marker genes for MC0 to MC3, respectively (Additional file 1: Table S5). To prioritise high-confidence cluster specific marker genes, we chose the top-ranked (by *P* value) 100 genes for each MC and performed a pathway enrichment analysis [41] (FDR-adjusted *P* < 0.05; Fisher’s exact test; Methods, Fig. 2b, Additional file 1: Table S6). MC1 was enriched for genes pertaining to pathways involving hypoxia, circadian clock, MAPK and MET signalling. MC2 was enriched for interferon (IFN)-related signalling, cell interaction, extracellular matrix organisation, and immunoregulatory interactions. MC3 was enriched for diseases of signal transduction by growth factor receptors, signalling by nuclear receptors, RNA polymerase III transcription, transcriptional regulation by RUNX3, ESR mediated, and Notch signalling. Notably, both MC1 and MC2 showed shared pathways related to interleukin signalling. Although this analysis failed to identify significantly enriched pathways for MC0, consideration of the top 200 marker genes identified weak enrichment of pathways related to energy metabolism and Notch signalling (Additional file 2: Fig. S3b). We further confirmed that key marker genes were expressed at the protein level through mass-spectrometry assessment of protein abundance (from Lei et al. [16]) and immunofluorescence (IF) staining of key markers and were associated with regions of hypoxia (CAIX positivity) (Additional file 2: Fig. S3c and Additional file 2: Fig. S4). We further confirmed the presence of these cell states in two syngeneic TNBC mouse models (4T1 and MMTV-PyMT) [59] (Additional file 2: Fig. S3d-e), indicating the validity of these cell states in the presence of an intact immune system.

We next performed the same analysis focussing on cycling S/G_2_M cells (Fig. 2c, Additional file 2: Fig. S5a-f, Methods). As expected, most MCs identified in S/G_2_M cells were enriched by cell cycle-related pathways, except for S/G_2_M-MC1, which was characterised by enrichment of hypoxia-related genes similarly to G_1_-MC1 (Additional file 2: Fig. S5e). To further decipher the link between G_1_ and S/G_2_M cells, we checked the common top 100 marker genes between G_1_ and S/G_2_M MCs (Fig. 2d). G_1_-MC1 and S/G_2_M-MC1 showed 73 out of 100 common marker genes, suggesting these two MCs may be the same cell states separated by non-proliferating and proliferating states.

### Distinct regulatory networks underpin cell state transcriptional regulation in TNBC residual disease

To further understand the higher-order regulation of transcriptional changes associated with the MCs, we applied SCENIC [42] to predict transcription factors (TF) activities and used AUCell, a regulon‑activity scoring method, to estimate TF activity (Methods). Although pathway enrichment analysis of marker genes of G_1_-MC0 did not reveal any significant pathways using the top 100 marker genes, the TF analysis identified 10 G_1_-MC0-specific TFs/TF regulators involved in development, and therefore we annotated G_1_-MC0 as a “developmental MC”, regulated by TFs including *ATF7*,* BCL11A*,* ERG*,* FOXN3*,* FOXP1*,* RARB*,* RUNX1*,* SOX5*,* STAU2*, and *TCF7L2* (Additional file 2: Fig. S6a). We also identified 17 G_1_-MC1-specific TFs/TF regulators functioning in hypoxia (*HIF1A*), stimuli or stress response (*CEBPG*,* HSF2*, and *MEF2A*), cell apoptotic and/or proliferation (*BCLAF1*,* DDIT3*,* FOS*, and *MAX*), circadian rhythm (*BHLHE40* and *NFIL3*), immune or tumour suppressor (*ANXA1*,* BACH2*,* CEBPD*, and *KLF6*), malignancies and tumorigenesis (*JUN* and *SOX4*), and epigenetic regulation (*KDM5B*), and S/G_2_M-MC-specific TFs whose functions are consistent with corresponding pathway analyses (Additional file 2: Fig. S6a-b). G_1_-MC2 and G_1_-MC3 did not reveal specific enrichment of TFs; however, these MCs did show enrichment of TFs shared with G_1_-MC0 and G_1_-MC1, highlighting likely shared higher-order regulatory mechanisms yet distinct downstream functional impact.

Specifically, *KDM5B* exhibited a significantly higher AUCell score in the hypoxia MCs (G_1_-MC1 *P* = 2.2 × 10^− 308^ and S/G_2_M-MC1 *P* = 2.1 × 10^− 270^, Kruskal–Wallis test; Additional file 2: Fig. S6c). Assessment of *KDM5B* gene expression levels and its TF regulon activity score in the single-cell dataset from Kim et al. 8 showed a significant enrichment in chemoresistant patients compared to chemosensitive patients’ pre-treatment biopsies and showed increased expression in post-NAC tumours in chemoresistant patients (Fig. 2e). KDM5B ChIP-seq data analysis also identified a significant enrichment of the marker genes pertaining to G_1_-MC1 that were bound at their promoters by KDM5B (Additional file 2: Fig. S6e-g). These observations suggest that KDM5B has higher epigenetic activity in chemoresistant patients and can be further induced by chemotherapy in resistant patients.

### The hypoxia cell state is enriched for “pre”-drug‑tolerant persister-like and metastatically primed cells

To examine the presence of known correlates of chemoresistance and disease aggressivity across these MCs, we tested a panel of previously identified gene expression signatures derived from both scRNA-seq and bulk RNA-profiling studies [7, 44–51] (Methods, Fig. 2f-i, Additional file 2: Fig. S7a-f). We first explored whether gene expression signatures associated with chromosomal instability (CIN70) [49], p53 status [50] (underlying genetic defects), and recombination proficiency (RPS [51], underlying DNA damage) were enriched in specific MCs (Methods, Fig. 2f-g, Additional file 2: Fig. S7a). We found that CIN70 was significantly higher in both G_1_-MC3 (*P* = 0.007) and S/G_2_M-MC3 (*P* < 0.001) whereas the p53 signature was significantly higher only in S/G_2_M-MC3 cells (*P* < 0.001), however remained suggestive of increased abundance in G_1_ cells (*P* = 0.054, Kruskal-Wallis tests; Fig. 2f-g). Therefore, we named these MCs as chromosomal instability and DNA damage-dominant MCs. Consistent with a previous report that shows cells with high rates of proliferation are more susceptible to DNA damage [71], the CIN70 signature score of cells in S/G_2_M phases were higher compared to those in G_1_ phase (Fig. 2f-g).

We found that significantly more cells in G_1_-MC1 (hypoxia) and G_1_-MC2 (IFN) showed detectable signatures (GSVA > 0) of drug-tolerant “persister-like” genes [7] compared to G_1_-MC0 (developmental) and G_1_-MC3 (chromosomal instability and DNA damage) (*P* < 0.001, Wilcoxon rank-sum test; Fig. 2h). Interestingly, many of these genes have been shown to be in a bivalent poised state [7] (*NDRG1*,* ABCA5*,* ZNF292*, and *NPAS2* from G_1_-MC1 and *RSAD2*,* CMPK2*,* CADPS2*,* TRANK1*,* LYN*,* PTPRM*,* MOXD1*, and *MAST4* from G_1_-MC2, Additional file 1: Table S7), i.e., the presence of both H3K4me3 activation and H3K27me3 repressive chromatin marks in treatment-naïve states, indicating the expression of these genes could be activated upon exposure to chemotherapy. We further found that S/G_2_M-MC1 (hypoxia) showed significantly higher detectable signatures of drug-tolerant “persister-like” genes (GSVA > 0) compared to other MCs (*P* < 0.001, Wilcoxon rank-sum test; Fig. 2i), suggesting the hypoxia subpopulation of persister cells were also proliferating. The IFN-like pre-DTP cell state was only evident in non-cycling cells suggestive of a population of drug-tolerant cells that have a blocked cell cycle. This finding is consistent with a recent study which reported cycling and non-cycling persister cells have antioxidant gene programmes and upregulation of IFN signatures, respectively [72]. As the post-NAC PDXs profiled are grown in the absence of therapy, these findings are consistent with a model of “pre-DTP” cells, or that these cells retain a “DTP memory” after initial chemotherapy exposure.

Previous studies have suggested that rare cells present in the primary tumour are transcriptionally “primed” to seed in distal tissues and cause metastases [46]. To ascertain if TNBC cells surviving post-NAC harbour metastatically primed–like properties, we applied known metastatic signatures including MBS, EMP, and a micrometastasis signature [44–46]. By applying these three signatures, we found that cells with high GSVA scores for EMP and micrometastasis signatures co-clustered in UMAP space (Additional file 2: Fig. S7b-c, Methods), indicating a subpopulation of cells that shared similar transcriptional programmes as metastatically primed cells (Additional file 2: Fig. S7e-f). Hypoxia MCs (G_1_-MC1 and S/G_2_M-MC1) contained the largest fraction of cells (GSVA > 0) expressing both the EMP and micrometastasis signatures compared to other MCs (Additional file 2: Fig. S7d). Together, these data suggest a population of “persister-like” cells are likely viable in hypoxic conditions and associated with the progression to distant metastatic disease.

### Post-NAC TNBC cell states are pre-existing in primary tumours and maintained in distant metastasis

**Fig. 3:**
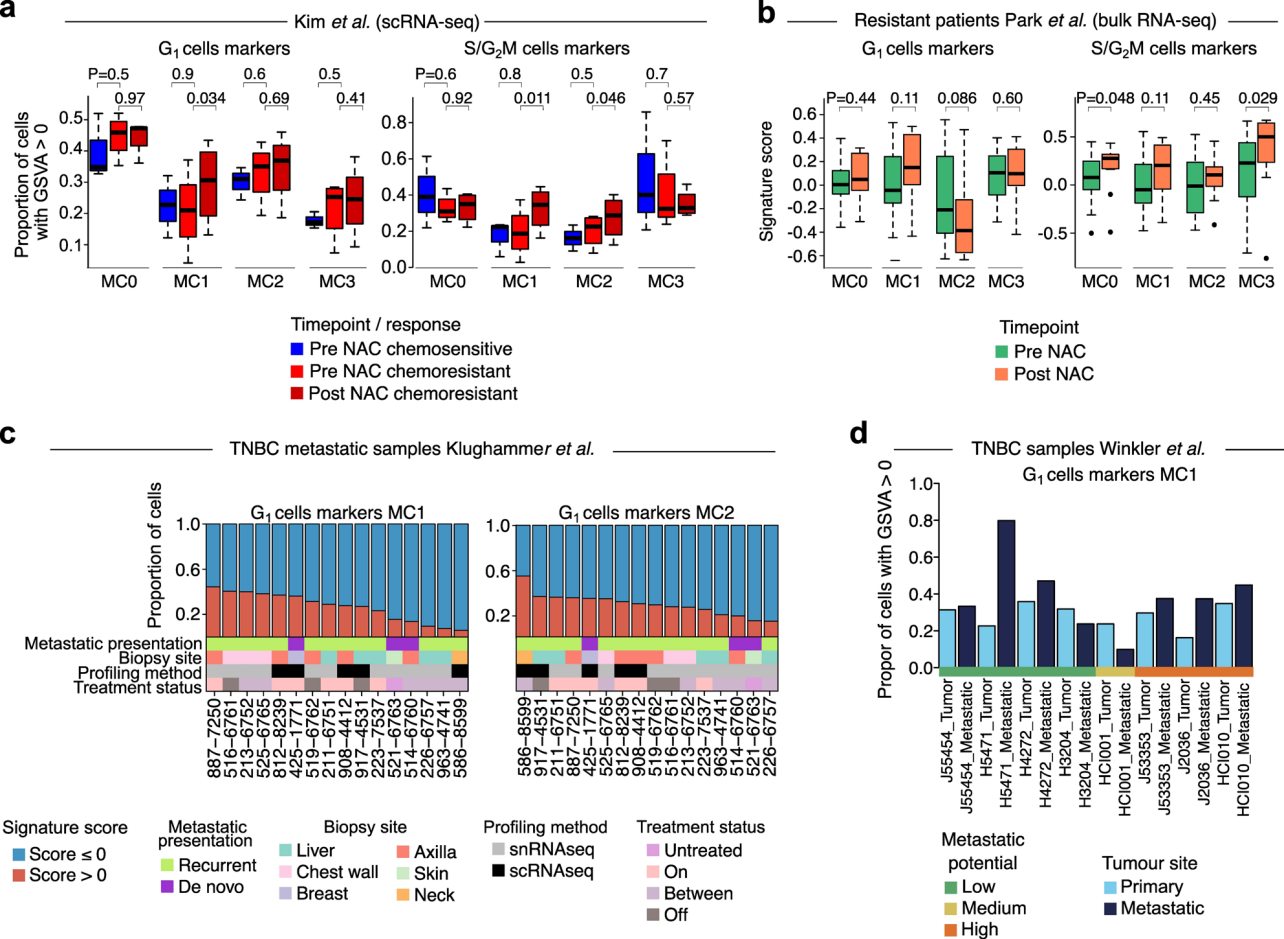
Subpopulations identified from residual disease are pre-existing in primary untreated tumours and are maintained in metastatic tumours. **a**. Box and whisker plots of proportion of cells expressing MC signatures (GSVA > 0) pre- and post-NAC in an independent single-cell dataset [8], showed significant enrichment of hypoxia MCs post-NAC in resistant patients (paired *t*-test). **b**. Box and whisker plots of signature scores (GSVA) of top 100 markers in resistant patients with pre- and post-NAC from an independent bulk dataset [64] on G_1_ (left) and S/G_2_M (right) cells MCs, showed enrichment of hypoxia MCs post-NAC (Wilcoxon rank-sum test). **c**. Bar plots of proportion of cells expressing MC signatures (GSVA > 0) of G_1_ cells MC1 (left) and MC2 (right) from residual disease in metastatic TNBC patients [56]. **d**. Bar plot of proportion of cells expressing G_1_-MC1 signature (GSVA > 0) in paired primary and metastatic PDX basal-like TNBC models from Winkler et al. [58], showing persister-like MC1 is maintained or enriched in metastatic disease.

To assess whether the subpopulations identified from our post-NAC PDX cohort are present in primary treatment-naïve TNBC and therefore are maintained and/or enriched post-treatment, we assessed their presence in an independent cohort of matched TNBC primary pre-treatment biopsies from chemotherapy-naïve tumours and post-treatment samples subjected to single-cell sequencing [8]. Patients with chemotherapy sensitive disease had pCR and no detectable cancer cells left in the breast, whereas patients with chemotherapy resistant disease had residual tumour present. Primary treatment-naïve TNBC showed a fraction of cells exhibiting characteristics of all MCs from our post-NAC PDX cohort (Fig. 3a), indicating their pre-existence. Specifically, patients with residual disease (i.e., chemoresistant) showed significant enrichment of cells exhibiting characteristics of persister-like hypoxia and IFN MCs G_1_-MC1 (*P* = 0.034, paired *t*-test), S/G_2_M-MC1 (*P* = 0.011, paired *t*-test), and S/G_2_M-MC2 (*P* = 0.046, paired *t*-test) post-NAC (Fig. 3a). Moreover, analysis of the single-cell data from pre-treatment biopsies from Kim et al. [8] showed that MCs harboured differential pathway activation between sensitive and resistant patients (Additional file 2: Fig. S8a-d), highlighting whilst these cell states pre-exist in treatment-naïve tumours, they also show some distinct characteristics. Using an independent RNA bulk dataset [64], we also observed that S/G_2_M-MC0 and S/G_2_M-MC3 showed enrichment post-NAC (*P* = 0.048 and *P* = 0.029, respectively, Wilcoxon rank-sum test on GSVA scores; Fig. 3b). We also found that some individual marker genes from the hypoxia MCs were significantly enriched post-NAC in the same bulk dataset (Additional file 1: Table S8). Notably, all MCs were found to pre-exist in treatment-naïve disease in the same bulk dataset indicated by individual marker genes (Additional file 1: Table S9). Together, these data suggest that chemotherapy resistant and DTP cells may already exist in treatment-naïve primary TNBC where they are primed to drive therapy resistance.

We next assessed the presence of cell states identified from our post-NAC PDX cohort in an independent cohort of metastatic tumours of TNBC subjected to single-cell sequencing [56]. Gene set scores of markers from our MCs clearly delineated these cell states in metastatic TNBC tumour cells (Fig. 3c and Additional file 2: Fig. S9a-b), suggesting that the cell states found in residual disease are maintained in metastatic tumours. This was further corroborated through analysis of matched primary and distant metastatic cells from a separate cohort of TNBC PDX models [58], which highlighted that the hypoxia cell state G_1_-MC1 and G_1_-MC2 were increased in the metastatic cells compared to the primary tumour in multiple PDXs (Fig. 3d, Additional file 2: Fig. S9c).

### Developmental and hypoxia cell states are predictive of a poor response to neoadjuvant chemotherapy

Next, we assessed whether the candidate resistant-associated MCs that are pre-existing in primary TNBC could be predictive of a poor response to NAC, and therefore, could potentially be used to identify patients harbouring therapy resistant cell states. To address this, we tested the gene sets (GSVA score) as well as the top 100 individual marker genes of our MCs in four independent cohorts by focusing on data from treatment-naïve TNBC patients, including BrighTNess [62], I-SPY2 [73] neoadjuvant clinical trials, Park et al. neoadjuvant chemotherapy study [64], and SCAN-B adjuvant chemotherapy treated TNBC cohort [60] (Methods, Additional file 1: Table S3).

**Fig. 4:**
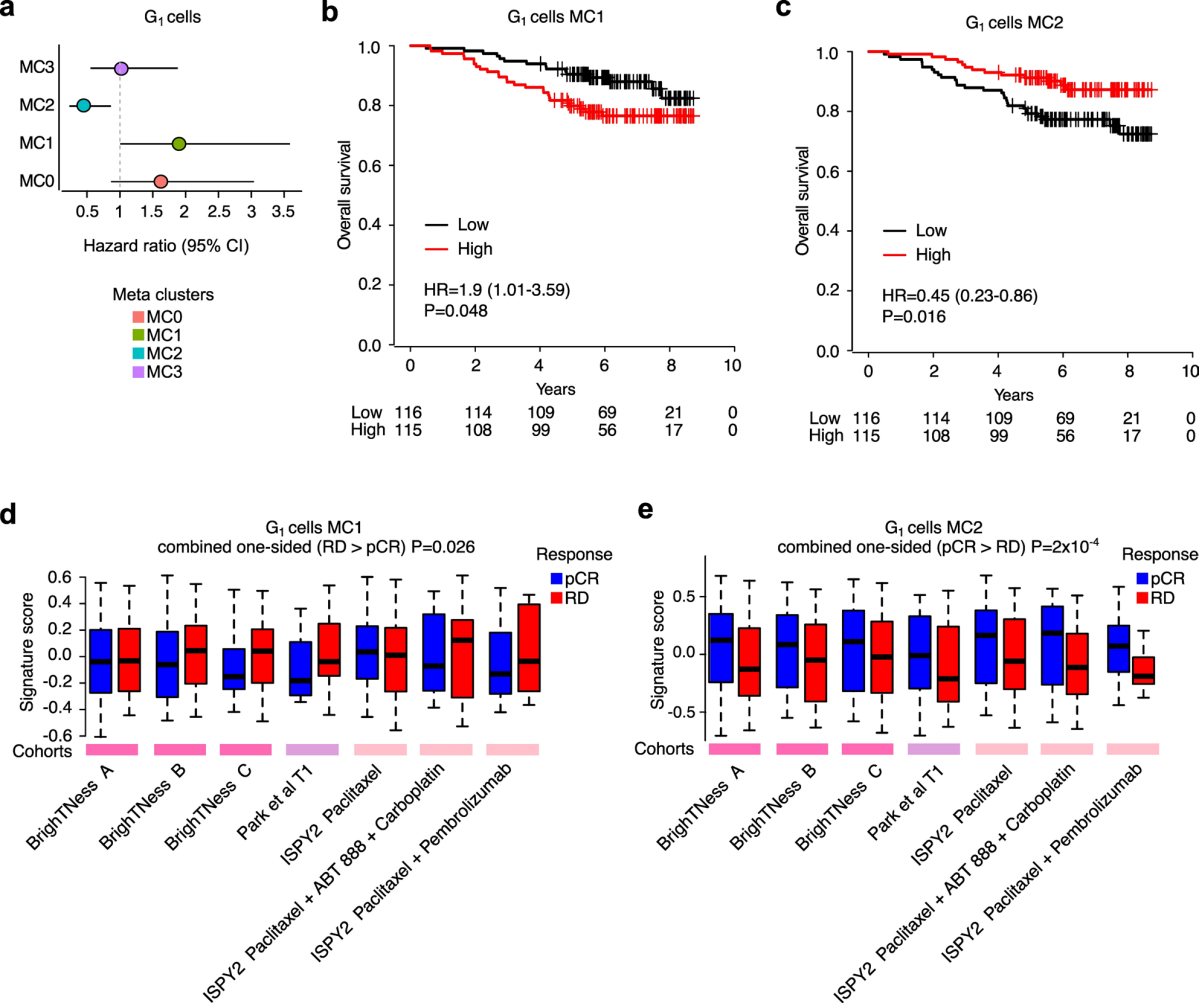
Developmental and hypoxia cell states are predictive of a poor response to neoadjuvant chemotherapy. **a**. Forest plot of HR of four MCs in G_1_ epithelial cells (GSVA score of top 100 marker genes) in SCAN-B [60] chemotherapy TNBC patients. Point represents treatment-specific HRs of MCs (as a continuous variable), error bar denotes 95% confidence interval (CI). **b-c**. Kaplan–Meier plots of G_1_ cells MC1 (**b**) and MC2 (**c**) GSVA score of top 100 marker genes in SCAN-B [60] chemotherapy TNBC patients showed significant association with poor and good prognosis, respectively, low: <= 50% quantile, high: > 50% quantile of GSVA score. **d-e**. Box and whisker plot of signature scores (GSVA score of top 100 marker genes) of chemo-naïve RD and pCR patients from seven arms with standard treatments (chemotherapy and/or immunotherapy) showed G_1_ cells MC1 markers (**d**) are expressed significantly higher in RD patients, while G_1_ cells MC2 markers (**e**) are expressed significantly higher in pCR patients (combined P of weighted sum of z method on one-sided Wilcoxon rank-sum test)

Quantifying marker genes of MCs as a per-patient score, we found the hypoxia MC (G_1_-MC1) was significantly associated with poor prognosis in the contemporary chemotherapy treated cohort SCAN-B (HR = 1.9, 95% CI = 1.01–3.59, *P* = 0.048), while the IFN signalling MC (G_1_-MC2) was significantly associated with good prognosis (HR = 0.45, 95% CI = 0.23–0.86, *P* = 0.016; Fig. 4a-c and Additional file 2: Fig. S10a). Assessment in pre-treatment biopsies from multiple neoadjuvant studies (Fig. 4d-e, Additional file 2: Fig. S10b, and Additional file 2: Fig. S11) highlighted that the developmental and hypoxia MCs (G_1_-MC0 and G_1_-MC1) exhibited significant enrichment in patients with RD (combined *P* = 0.026, sum of z method on one-sided Wilcoxon rank-sum test; Fig. 4d and Additional file 2: Fig. S10b), while the IFN signalling MC (G_1_-MC2) exhibited significant enrichment in those patients with pCR (combined *P* = 2 × 10^− 4^, sum of z method on one-sided Wilcoxon rank-sum test; Fig. 4e).

### KDM5 inhibition suppresses the emergence of drug‑tolerant persister cells *in vitro*

**Fig. 5:**
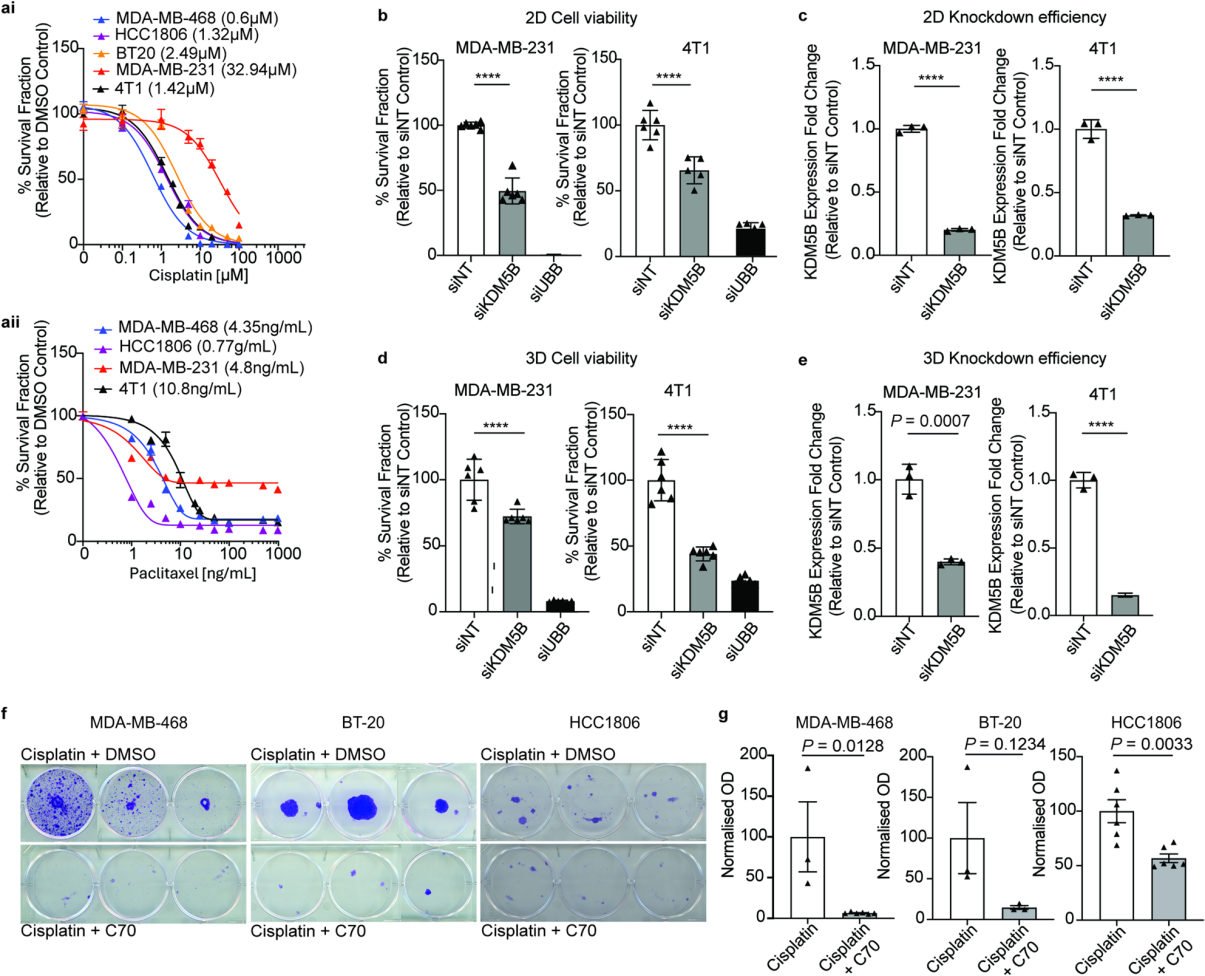
KDM5B inhibition delays drug-tolerant persister cell recovery post chemotherapy exposure. **a**. Cisplatin and paclitaxel dose response of TNBC cell lines and 4T1 murine cells where proliferation was assessed by CellTiter-Glo assay after 72 h. The drug concentration that inhibits the growth of 50% of cells is denoted in brackets. **b**. 72 h 2D viability assay assessed by CellTitre-Glo cell viability assay of MDA-MB-231 (left) and 4T1 (right) cells after transfection with non-targeting control (siNT), KDM5B or UBB (positive killing control) siRNA (20 nM). ****P < 0.0001 one-way ANOVA with Bonferroni’s multiple comparison test, 6–12 replicates. **c**. Quantitative PCR of *KDM5B* mRNA expression of MDA-MB-231 (left) and 4T1 (right) cells after 72 h siNT or KDM5B siRNA in 2D. **d**. 7-day spheroid assay assessed by Alamar Blue cell viability stain of MDA-MB-231 (left) and 4T1 (right) cells after transfection with non-targeting control, KDM5B or UBB (positive killing control) siRNA (20 nM). ****P < 0.0001 (one-way ANOVA with Bonferroni’s multiple comparison test, 6–12 replicates/treatment. **e**. Quantitative PCR of *KDM5B* mRNA expression of MDA-MB-231 (left) and 4T1 (right) cells after 72 h siNT or KDM5B siRNA in 3D spheroids. **f**. Colony forming assays were performed on MDA-MB-468 (left), BT-20 (middle), and HCC1806 cells (right) treated with cisplatin (IC50 of that cell line) for 72 h (top) followed by C70 (30 μM) for 3 days (bottom). Cells were washed at 3 and 6 days post cisplatin treatment. Colonies were fixed and stained with crystal violet once colonies appeared in the Cisplatin + C70 treated wells (days 18–44). **g**. Crystal violet stained colonies from (**h**) were solubilised as a readout of colony recovery, where optical density (OD) is a measure of cell biomass, for MDA-MB-468 (left), BT-20 (middle), and HCC1806 cells (right) treated with cisplatin with and without C70 (n = 3–6 replicates/drug exposure, unpaired *t*-test)

Given our observed correlation between the hypoxia cell states G_1_-MC1 and S/G_2_M-MC1 with features of DTP cells, as proof-of-concept, we sought to functionally test the effects of perturbing the KDM5B TF on cell viability in cells inherently less sensitive to commonly used chemotherapies (Fig. 5a). siRNA-mediated KDM5B knockdown led to a significant reduction in cell viability compared to non-targeting (siNT controls) in both 2D and 3D spheroid culture systems in human (MDA-MB-231) and murine models (4T1) of TNBC (Fig. 5b-e). Furthermore, exposure of the chemotherapy sensitive TNBC cell lines BT-20, HCC1806, and MDA-MB-468 cells to a single dose of cisplatin for 72 h followed by 3-day exposure to the KDM5 inhibitor C70 significantly delayed the colony-forming ability of cells compared to DMSO controls (Fig. 5f-g).

Together, these data highlight that developmental and hypoxia cell states present in residual disease may drive TNBC neoadjuvant and adjuvant chemotherapy resistance, however this warrants further functional *in vitro* and *in vivo* assessment to assume causality.

## Discussion

In this study, we created a high-depth, high-resolution atlas of fourteen PDX models derived from TNBC primary tumours with viable residual cancer post-NAC using snRNA-seq. Using an integrative meta clustering approach focussing on G_1_ and S/G_2_M phases separately, we identified heterogeneous transcriptionally defined subpopulations (cell states) of cells associated with therapy resistance and metastasis that are pre-existing in therapy-naïve tumours, are enriched in residual disease, maintained at distant metastatic sites, and are predictive for chemotherapy response and overall prognosis in TNBC. These data are highly suggestive of a model where these cell states are stably selected for upon chemotherapy exposure and subsequently inherited.

By performing pathway enrichment and higher-order TF inference analysis and applying published breast cancer signatures, we identified and validated the presence of cell states enriched for developmental, hypoxia, IFN signalling, and chromosomal instability and DNA damage associated pathways. G_1_-MC1 and S/G_2_M-MC1 show activated pathways of hypoxia, circadian clock, MAPK and MET signalling, which have been implicated in driving cancer progression [74–77]. Moreover, a small population of cells from the hypoxia MC were found to be enriched for signatures identified in distant metastatic cells. G_1_-MC2 showed IFN signalling-related pathways which are associated with immunomodulatory signalling, indicating its anti-tumourigenic properties [78]. We found that both the hypoxia and IFN signalling MCs exhibit “persister-like” features that were previously identified from cells remaining post chemotherapy (5-FU) exposure in TNBC that were found to be enriched for poised or bivalent genes harbouring H3K4me3 promoter activation and H3K27me3 repressive marks. Bivalency is a feature of stem cells that allows quick transition between different cell states [7]. Here we validated the presence of the hypoxia and IFN signalling cell states in treatment-naïve TNBC patients from an independent cohort [8]. Indeed, the study from Marsollier et al. found that suppression of the H3K27me3 repressive mark in combination with chemotherapy could reverse the persister cell state and render cells sensitive to chemotherapy [7]. Here, we illustrate that some of the genes that are bivalent in primary disease persist and are actively proliferating in tumours derived from patients post-NAC. This suggests that they retain “DTP memory” and highlights their role in stabilising a therapy resistant state. Interestingly, hypoxia has been shown to increase epigenetic bivalency which overlaps with embryonic stem cell-associated genetic bivalency [79]. This is in line with our findings that the hypoxia and “persister-like” cells coexist in the same MC or cell state. Previous studies have shown that tumour cells can exhibit reversible tolerance to drugs resulting in a reversal of cell states to that seen in primary disease [80]. In the models characterised herein, we observed that some showed resistance to docetaxel and/or carboplatin when rechallenged *in vivo* (as described in [16] although we appreciate these maybe sub-maximal doses*)*, with only one model BCM-0104, showing a complete response (i.e. non-palpable tumour in all animals). Notably, the patient from which this model was derived had a pCR and was alive at the time of last follow up, whereas all other patients had died of their disease. The presence of the persister-like MCs in these models suggests that some of marker genes of these MCs are activated under chemotherapy exposure through suppression of H3K27me3 and maintain their active status to drive stable resistance, even in the absence of therapy.

Higher expression of antioxidant gene programmes and a metabolic shift to fatty acid and the upregulation of IFN signatures have been reported as properties of cycling and non-cycling persister cells [72]. Based on the cell cycle state and the enrichment of IFN signalling pathway, we hypothesised that our hypoxia MCs (G_1_-MC1 and S/G_2_M-MC1) and IFN signalling MC (G_1_-MC2) matched the cycling and non-cycling persister cells. Corroborating these observations, we found that the IFN signalling MC (G_1_-MC2) was associated with good prognosis, while the hypoxia (G_1_-MC1 and S/G_2_M-MC1) and the developmental MC (G_1_-MC0) were associated with poor prognosis in chemotherapy-treated TNBC patients. Markers from developmental and hypoxia MCs were found to be significantly associated with chemotherapy resistance and/or poor prognosis in four independent cohorts (*NIPAL2*,* MAML3*,* ADCY2*,* DMD*,* SPIDR*,* PKN2-AS1*,* ARFGEF3*,* EMP1*,* KDM5B*,* NEAT1*,* ANKRD37*,* HBP1*,* HK2*, and *RYR2*) and enriched in resistant samples post-chemotherapy in an independent single-cell dataset [8] (*ANKRD37*,* KDM5B*,* RYR2*, and *ADCY2*). Many of these genes have been reported to be associated with development of cancer, drug resistance or metastasis [81–86]. The epigenetic regulator *KDM5B* has been demonstrated to lead to higher transcriptomic heterogeneity and poor prognosis in ER+ve disease [65] and is known to promote immune evasion [87]. Here, we show that *KDM5B* is a potential master regulator of the hypoxia cell state and show increased KDM5B binding to the promoters of G_1_-MC1 marker genes. We further demonstrate using data from an independent single-cell cohort [8] that chemotherapy resistant patients have significantly higher *KDM5B* regulon activity compared to sensitive patients, and this activity increases post-therapy in resistant patients. Moreover, pharmacological inhibition of KDM5 activity delays the colony-forming ability of multiple TNBC cell lines after exposure to a single dose of cisplatin. This highlights a model whereby master regulation of this cell state is pre-existing but is also further induced upon chemotherapy exposure and is stably inherited to allow maintenance of this activity at distant metastatic sites.

Our study has some limitations. Firstly, as we performed snRNA-seq, cytoplasmic RNA was not captured. Secondly, we do not have matched primary patient single-cell data to confirm the pre-existence of cell states characterised from the PDX models and hence were unable to perform trajectory analyses. Thirdly, the patients subjected to snRNA-seq from Kim et al. [8] were additionally treated with the experimental drug bevacizumab, which could have introduced artefacts. Additionally, our sample response groups are imbalanced, thus we cannot confidently find chemosensitive-specific properties. Evaluating marker genes identified from our single-nuclei experiments based on cancer cells on bulk RNA-seq datasets that contain heterogeneous cell types to find association with resistance and survival is another limitation. We also acknowledge lack of protein validation of our cell states in metastatic sites of the PDX models used in this study.

## Conclusions

In summary, we have characterised the transcriptional diversity and chemoresistance-associated subclonal composition of a large panel of PDX models generated from patients with residual disease post-NAC. We identified cell states marked by hypoxia signatures that are enriched for high expression of “persister-like” gene programmes that are both prognostic and predictive of poor therapy response in untreated TNBC. These cell states confer transcriptional plasticity and survival advantages under therapeutic pressure, suggesting that chemotherapy selects for, rather than induces these programmes. Furthermore, we found a subset of these genes is likely to be stably inherited and drive resistance to multiple types of chemotherapy and warrant further validation as both potential biomarkers and as new therapeutic targets. Collectively, these findings highlight the importance of non‑genetic resistance mechanisms and propose new opportunities for therapeutic intervention in triple‑negative breast cancer. This dataset provides a valuable resource for functional interrogation of the subclonal heterogeneity of post-NAC TNBC at high single-nuclei resolution.

## Supplementary Information

Additional file 1. Table S1. Patient information and PDX *in vivo* response. Table S2. Quality control and filters in snRNA-seq samples. Table S3. Clinical trials or cohorts used for treatment response and prognosis prediction. Table S4. Filtered variants on COSMIC cancer gene census from seven PDX models. Table S5. Significant marker genes for meta clusters. Ranked by the combined *P*. Table S6. Significant pathways for meta clusters. Table S7. Genes in bivalent chromatin configuration [7] in chemo-naïve cells locked by H3K27me3 from the top 100 marker genes of meta clusters. Table S8. Significantly enriched markers post-treatment in resistant patients from a bulk dataset [64] of hypoxia meta clusters. One-sided Wilcoxon ranksum test, *P* < 0.05. Table S9. Pre-existing markers in resistant patients from a bulk dataset [64], for which the median of log_2_(TPM) × tumour purity in all pre-treatment resistant patients > log_2_(5).

Additional file 2. Supplementary figures Fig. S1–S11.

## Data Availability

Raw single-cell RNA-seq data generated during the current study are available in the SRA repository, under accession number PRJNA1441590. The authors confirm that data processing and analysis used existing code/software packages whose details are provided in the Methods. An interactive web app [88] was developed to visualise processed data at: https://software.icr.ac.uk/app/bcsinglecellalmanac-pdx-nac. The public scRNA-seq data of pre- and post-NAC patient cohort was obtained from SRA: SRP114962 [8]. The public sc(sn)RNA-seq data of metastatic patient cohort was obtained from CELLxGENE website. The public matched primary and metastatic PDX cohort was retrieved from GEO: GSE210283 [59]. Therapy response clinical cohorts were sourced as follows: BrighTNess from GEO: GSE164458 [62], I-SPY2 from GEO: GSE194040 [63], and Park et al. cohort from GEO: GSE123845 [64]. SCAN-B survival clinical cohort was downloaded from Mendeley Data [61]. Public KDM5B ChIP-seq data was retrieved from GEO under accession number GSE46055 [65]. Public proteomics data of TNBC PDXs were retrieved from Lei et al. [16].

## Abbreviations

TNBC: Triple‑negative breast cancer
NAC: Neoadjuvant chemotherapy
PDX: Patient‑derived xenograft
IFN: Interferon signalling
RCB: Residual cancer burden
CNA: Copy number alteration
H3K27me3: Histone H3 lysine‑27 trimethylation
H3K4me3: Histone H3 lysine‑4 trimethylation
snRNA‑seq: Single‑nucleus RNA sequencing
BCM: Baylor College of Medicine
HCI: Huntsman Cancer Institute
JAX: The Jackson Laboratory
IACUC: Institutional Animal Care and Use Committee
STR: Short tandem repeat
WES: Whole exome sequencing
FC: Fold change
SNV: Single nucleotide variant
FACS: Fluorescence‑activated cell sorting
IF: Immunofluorescence
IHC: Immunohistochemistry
SD: Standard deviation
UMI: Unique molecular identifier
RPL: Ribosomal protein large subunit
PC: Principal component
PCA: Principal component analysis
UMAP: Uniform Manifold Approximation and Projection
MC: Meta cluster
HVG: Highly variable gene
FDR: False discovery rate
OC: Overlap coefficient
GSVA: Gene set variation analysis
SCENIC: Single–cell regulatory network inference and clustering
GRN: Gene regulatory network
TF: Transcription factor
AUCell: AUC-based regulon activity scoring
MBS: Metastatic burden signature
EMP: Epithelial-mesenchymal plasticity
RTS: Residual tumour signature
PS: Prognostic signature
CIN70: Chromosomal instability 70‑gene signature
RPS: Recombination proficiency score
qPCR: Quantitative PCR
BC: Breast cancer
SCAN‑B: Sweden Cancerome Analysis Network–Breast cohort
HR: Hazard ratio
RD: Residual disease
pCR: Pathological complete response
FFPE: Formalin‑fixed paraffin‑embedded tissue
ChIP‑seq: Chromatin immunoprecipitation sequencing
NT: Non–targeting
GEO: Gene Expression Omnibus
DTP: Drug‑tolerant persister
