# Supplementary material for: Co-occurrence of transcriptionally distinct persister cell states underpins neoadjuvant therapy resistance in triple‑negative breast cancer
Source: Genome Med. 2026 Apr 9;18:65. doi: 10.1186/s13073-026-01643-9 (PMC13173903; doi:10.1186/s13073-026-01643-9)
Supplement: Supplementary file 2 — Additional file 2. Supplementary figures Fig. S1–S11. [file 13073_2026_1643_MOESM2_ESM.pdf]

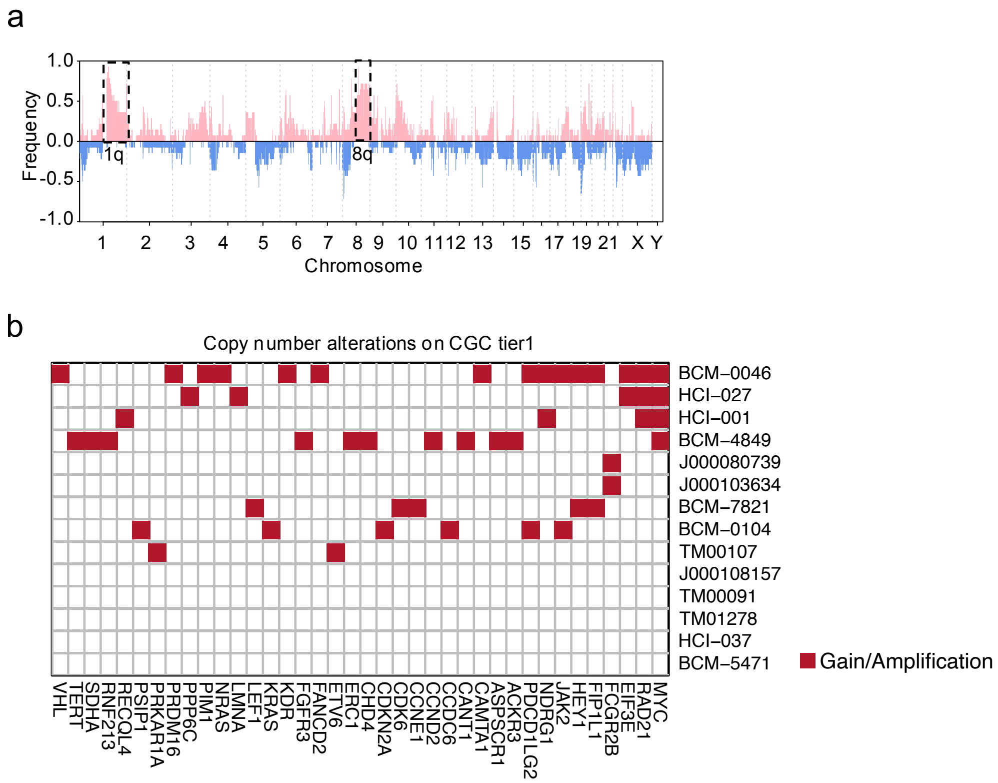

Fig.S1. Copy number alternations (CNA) in 14 PDX samples. a. Frequency of CNA from 14 samples showed typical TNBC CNA gain on chromosome 1q and 8q. **b.** CNA on each sample of CGC tier 1 gene list. The Gain/Amplification represents that gene has $\log_2$(FC ratio)/standard deviation (SD) > 3 in corresponding samples.

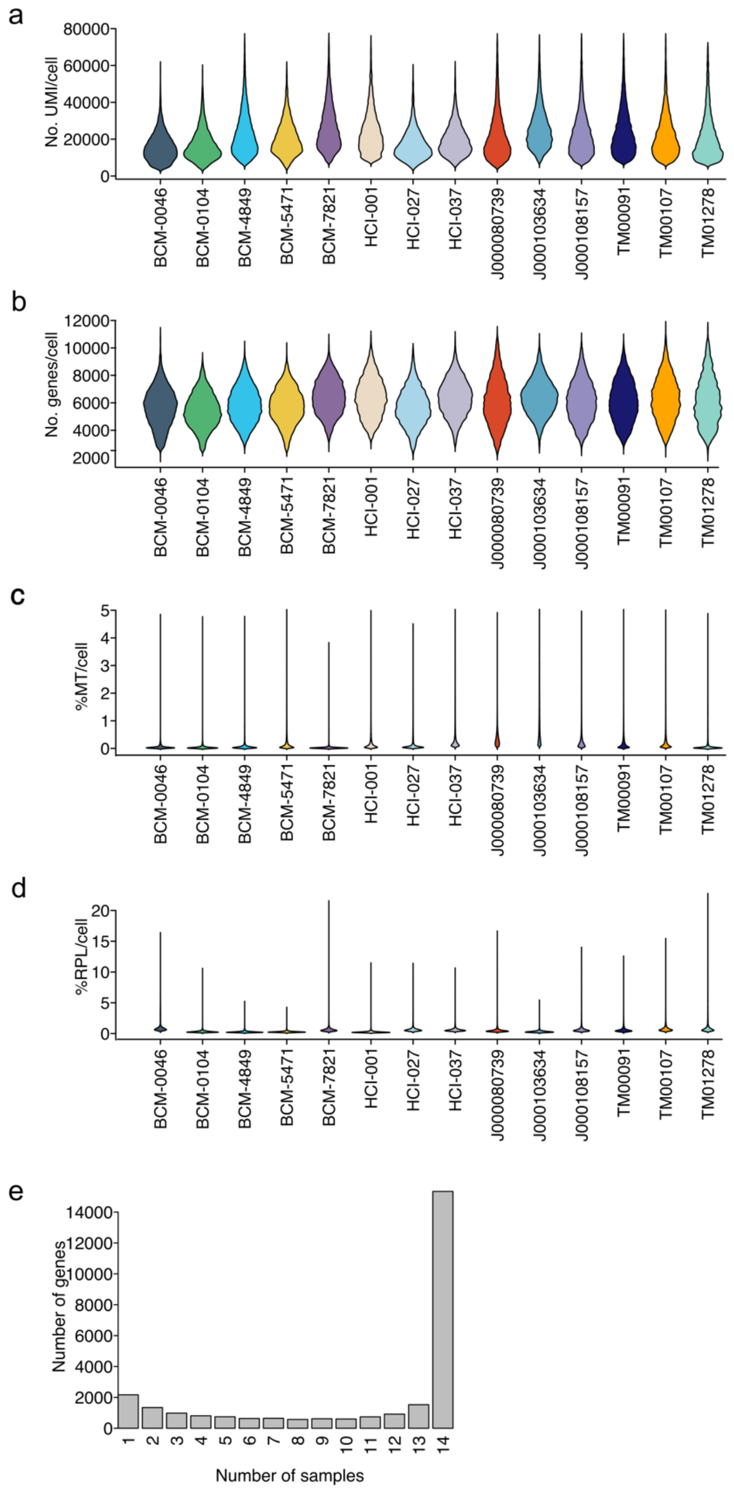

Fig.S2. Quality control and inter tumour heterogeneity. a-d. Violin plots on cells passed the quality control for 14 samples of **a.** number of reads per cell, **b.** number of genes per cell, **c.** percentage of mitochondrial reads per cell, and **d.** percentage of ribosomal reads per cell. **e.** Number of genes that are common in 1 to 14 PDXs.

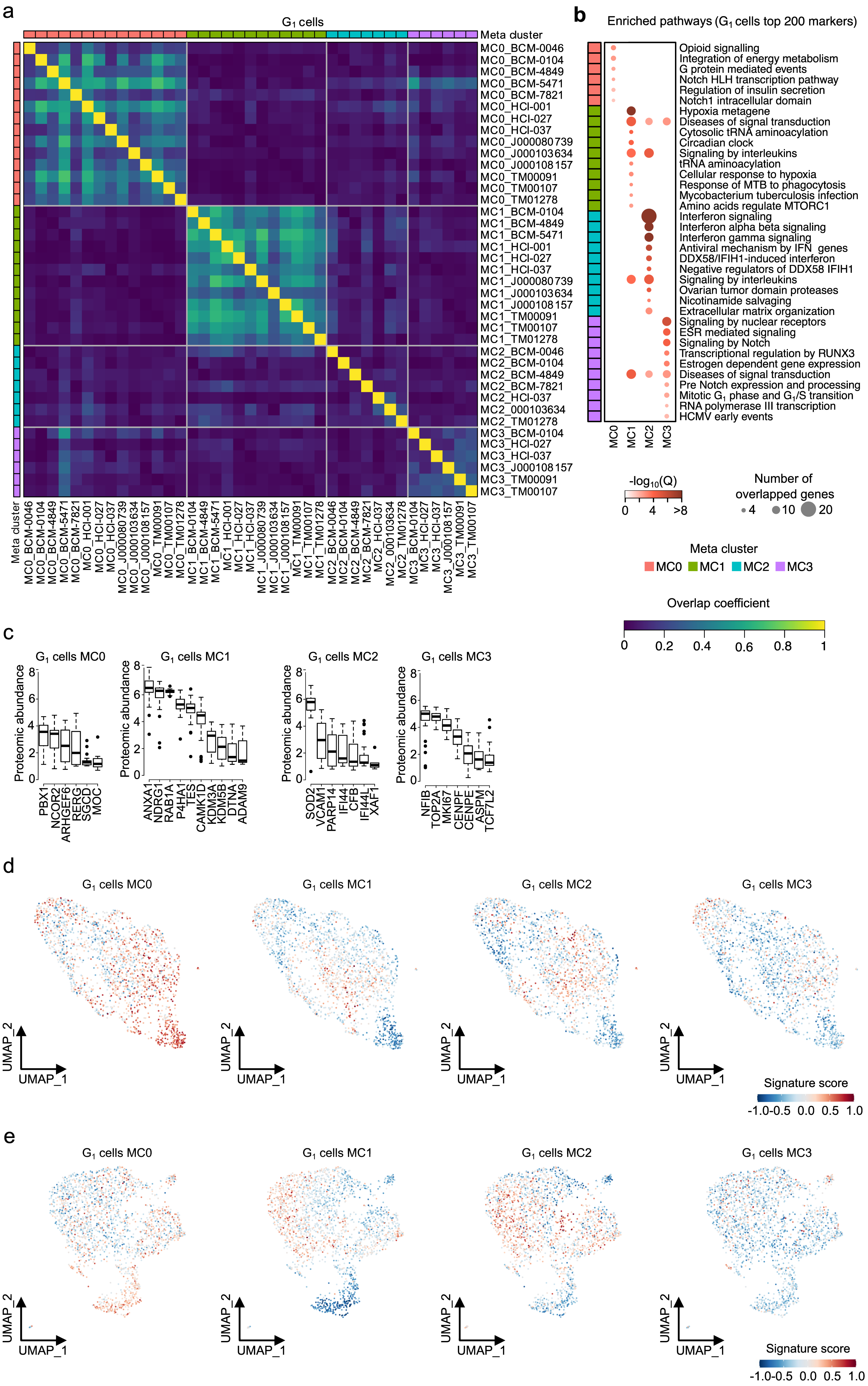
a
G1 cells
Meta cluster
MC0_BCM-0046
MC0_BCM-0104
MC0_BCM-4849
MC0_BCM-5471
MC0_BCM-7821
MC0_HCI-001
MC0_HCI-027
MC0_HCI-037
MC0_J000080739
MC0_J000103634
MC0_J000108157
MC0_TM00091
MC0_TM00107
MC0_TM01278
MC1_BCM-0104
MC1_BCM-4849
MC1_BCM-5471
MC1_HCI-001
MC1_HCI-027
MC1_HCI-037
MC1_J000080739
MC1_J000103634
MC1_J000108157
MC1_TM00091
MC1_TM00107
MC1_TM01278
MC2_BCM-0046
MC2_BCM-0104
MC2_BCM-4849
MC2_BCM-7821
MC2_HCI-037
MC2_000103634
MC2_TM01278
MC3_BCM-0104
MC3_HCI-027
MC3_HCI-037
MC3_J000108157
MC3_TM00091
MC3_TM00107
b
Enriched pathways (G1 cells top 200 markers)
Opioid signalling
Integration of energy metabolism
G protein mediated events
Notch HLH transcription pathway
Regulation of insulin secretion
Notch1 intracellular domain
Hypoxia metagene
Diseases of signal transduction
Cytosolic tRNA aminoacylation
Circadian clock
Signaling by interleukins
tRNA aminoacylation
Cellular response to hypoxia
Response of MTB to phagocytosis
Mycobacterium tuberculosis infection
Amino acids regulate MTORC1
Interferon signaling
Interferon alpha beta signaling
Interferon gamma signaling
Antiviral mechanism by IFN genes
DDX58/IFIH1-induced interferon
Negative regulators of DDX58 IFIH1
Signaling by interleukins
Ovarian tumor domain proteases
Nicotinamide salvaging
Extracellular matrix organization
Signaling by nuclear receptors
ESR mediated signaling
Signaling by Notch
Transcriptional regulation by RUNX3
Estrogen dependent gene expression
Diseases of signal transduction
Pre Notch expression and processing
Mitotic G1 phase and G1/S transition
RNA polymerase III transcription
HCMV early events
MC0 MC1 MC2 MC3
-log10(Q)
0 4 >8
Number of overlapped genes
4 10 20
Meta cluster
MC0 MC1 MC2 MC3
Overlap coefficient
0 0.2 0.4 0.6 0.8 1
c
G1 cells MC0
Proteomic abundance
PBX1 NCOR2 ARHGEF6 RERG SGCD MOC
G1 cells MC1
ANXA1 NDRG1 RAB1A P4HA1 TES CAMK1D KDM3A KDM5B DTNA ADAM9
G1 cells MC2
SOD2 VCAM1 PARP14 IFI44 CFB IFI44L XAF1
G1 cells MC3
NFIB TOP2A MKI67 CENPF CENPE ASPM TCF7L2
d
G1 cells MC0
G1 cells MC1
G1 cells MC2
G1 cells MC3
UMAP_1
UMAP_2
Signature score
-1.0 -0.5 0.0 0.5 1.0
e
G1 cells MC0
G1 cells MC1
G1 cells MC2
G1 cells MC3
UMAP_1
UMAP_2
Signature score
-1.0 -0.5 0.0 0.5 1.0

Fig.S3. Post NAC TNBC harbour transcriptionally defined cell states. a. Heatmap of the overlapping coefficient between the significant differentially expressed genes (DEGs) on every two subclusters from G_1 epithelial cells, indicating the DEGs from different samples but in the same meta clusters (MC) share similarities. The subcluster is defined as cells belonging to the same MC within each sample. **b.** Pathway enrichment for all or top 10 most significant pathways in G_1 epithelial cells from Reactome database in MSigDB based on top 200 marker genes. The colour of dot represents $-\log_{10}$(FDR-adjusted *P*) and the size of dot represents the number of genes intersected by top 200 marker genes and pathway gene sets. **c**. Box plots of the top marker genes from G_1-MC1 and G_1-MC2 detected by mass spectrometry analysis of TNBC PDX models. Y-axis is the $\log_{10}$-transformed, batch corrected, iBAQ intensities. **d-e**. UMAP visualisation of signature score (GSVA) of top 100 marker genes from G_1-MCs in $EpCAM^+$ epithelial cells 4T1(d) and MMTV-PyMT (e) TNBC murine models. GSVA scores were scaled to [-1,1].

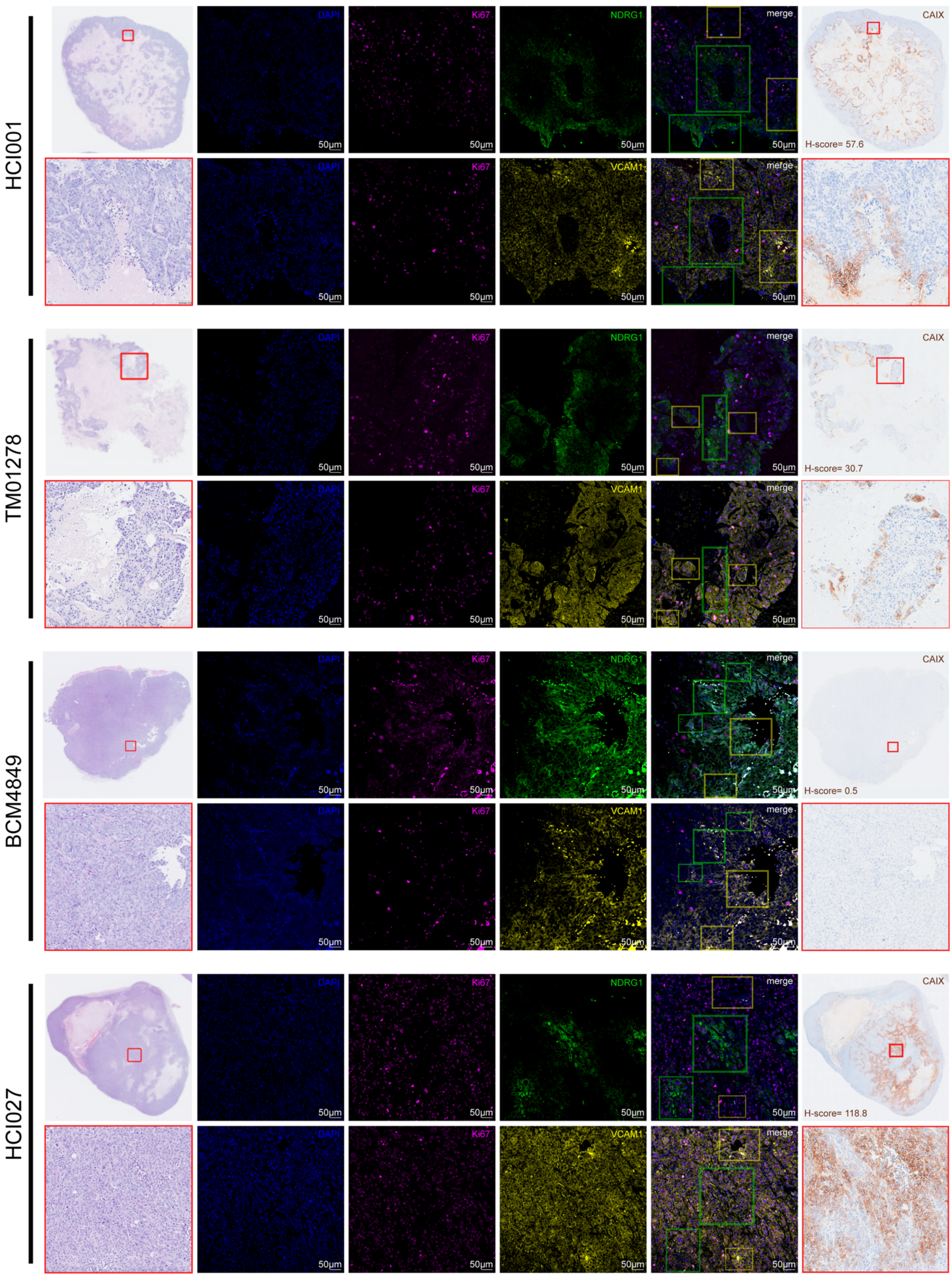

Fig.S4. **Cell state markers are expressed at the protein level.** a-d. Protein level expression of the hypoxia marker CAIX detected by immunohistochemistry and protein level expression of G1-MC1 marker NDRG1 and G1-MC2 marker VCAM1 in representative TNBC PDXs. Serial sections were stained for Ki67 proliferation marker (magenta) in combination with either NDRG1 marking MC1 cells (green) or VCAM1 marking MC2 cells (yellow). DAPI was used as counterstain to visualise nuclei. Whole slide haematoxylin and eosin staining is provided for general tumour architecture, highlighting the representative areas (red square) that were captured. Immunohistochemistry staining of carbonic anhydrase IX was performed on a separate section

to mark areas of increased hypoxia and to quantify hypoxia in individual PDXs. Areas of higher expression of MC1 or MC2 markers are outlined on merged multiplex images using respectively coloured boxes. Scalebar depicts 50μm.

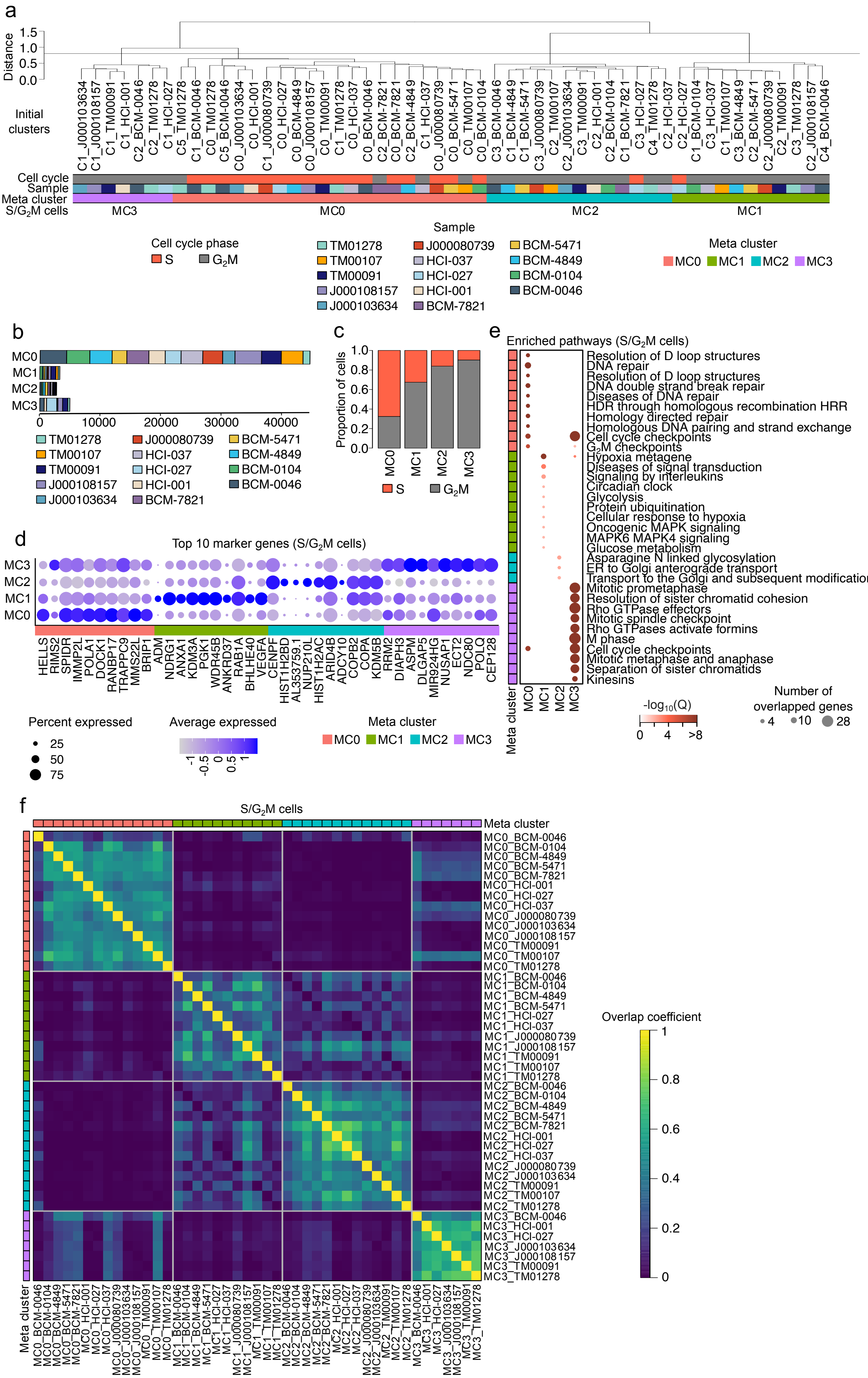
a
Distance
1.5
1.0
0.5
0.0
Initial clusters
Cell cycle
Sample
Meta cluster
S/G2M cells
MC3
MC0
MC2
MC1
Sample
Cell cycle phase
S
G2M
TM01278
TM00107
TM00091
J000108157
J000103634
J000080739
HCI-037
HCI-027
HCI-001
BCM-7821
BCM-5471
BCM-4849
BCM-0104
BCM-0046
Meta cluster
MC0
MC1
MC2
MC3
b
MC0
MC1
MC2
MC3
0
10000
20000
30000
40000
c
Proportion of cells
1.0
0.8
0.6
0.4
0.2
0.0
MC0
MC1
MC2
MC3
S
G2M
d
Top 10 marker genes (S/G2M cells)
HELLS
RIMS2
SPIDR
IMMP2L
POLA1
DOCK1
RANBP17
TRAPPC9
MMS22L
BRIP1
ADM
NDRG1
ANXA1
KDM3A
PGK1
WDR45B
ANKRD37
RAB1A
BHLHE40
VEGFA
CENPF
HIST1H2BD
AL353759.1
NUP210L
HIST1H2AC
ARID4B
ADCY10
COPB2
COPA
KDM5B
RRM2
DIAPH3
ASPM
DLGAP5
MIR924HG
NUSAP1
ECT2
NDC80
POLQ
CEP128
Percent expressed
25
50
75
Average expressed
-1
-0.5
0
0.5
1
Meta cluster
e
Enriched pathways (S/G2M cells)
Resolution of D loop structures
DNA repair
Resolution of D loop structures
DNA double strand break repair
Diseases of DNA repair
HDR through homologous recombination HRR
Homology directed repair
Homologous DNA pairing and strand exchange
Cell cycle checkpoints
G2M checkpoints
Hypoxia metagene
Diseases of signal transduction
Signaling by interleukins
Circadian clock
Glycolysis
Protein ubiquitination
Cellular response to hypoxia
Oncogenic MAPK signaling
MAPK6 MAPK4 signaling
Glucose metabolism
Asparagine N linked glycosylation
ER to Golgi anterograde transport
Transport to the Golgi and subsequent modification
Mitotic prometaphase
Resolution of sister chromatid cohesion
Rho GTPase effectors
Mitotic spindle checkpoint
Rho GTPases activate formins
M phase
Cell cycle checkpoints
Mitotic metaphase and anaphase
Separation of sister chromatids
Kinesins
Meta cluster
MC0
MC1
MC2
MC3
-log10(Q)
0
4
>8
Number of overlapped genes
4
10
28
f
S/G2M cells
Meta cluster
MC0_BCM-0046
MC0_BCM-0104
MC0_BCM-4849
MC0_BCM-5471
MC0_BCM-7821
MC0_HCI-001
MC0_HCI-027
MC0_HCI-037
MC0_J000080739
MC0_J000103634
MC0_J000108157
MC0_TM00091
MC0_TM00107
MC0_TM01278
MC1_BCM-0046
MC1_BCM-0104
MC1_BCM-4849
MC1_BCM-5471
MC1_HCI-027
MC1_HCI-037
MC1_J000080739
MC1_J000108157
MC1_TM00091
MC1_TM00107
MC1_TM01278
MC2_BCM-0046
MC2_BCM-0104
MC2_BCM-4849
MC2_BCM-5471
MC2_BCM-7821
MC2_HCI-001
MC2_HCI-027
MC2_HCI-037
MC2_J000080739
MC2_J000103634
MC2_TM00091
MC2_TM00107
MC2_TM01278
MC3_BCM-0046
MC3_HCI-001
MC3_HCI-027
MC3_J000103634
MC3_J000108157
MC3_TM00091
MC3_TM01278
Overlap coefficient
1
0.8
0.6
0.4
0.2
0

[1]**Fig.S5. Cells in S/G_2M phases from post NAC TNBC harbour transcriptionally defined cell states. a.** Dendrogram of meta clustering on S/G_2M epithelial cells from the 14 PDX models. (C#_ indicates the cluster number when the initial clustering was performed on the individual PDXs). The meta clusters (MCs) were determined at cut-off = 0.8. Initial clusters were obtained at optimal resolution defined by Davies–Bouldin index within each sample. Note phase refers to the cell cycle phase (S-red and G_2M- grey). **b.** Number of S/G_2M cells from each sample in each MC. **c.** Proportion of cells in S (red) and G_2M (grey) cell cycle phases for each sample. **d.** Dot plot of top 100 MC marker genes of S/G_2M epithelial cells show MC specificity. The colour of the dot represents average gene expression, and the size of the dot represents percentage of cells expressing the gene. **e.** Pathway enrichment for all or top 10 most significant pathways in S/G_2M cells from Reactome database in MSigDB. The colour of dot represents -$\log_{10}$(FDR-adjusted *P*) and the size of dot represents the number of genes intersected by top 100 marker genes and pathway gene sets. **f.** Heatmap of the overlap coefficient between the significant DEGs on every two subclusters from S/G_2M epithelial cells, indicating the DEGs from different samples but in the same meta clusters (MC) share similarities.

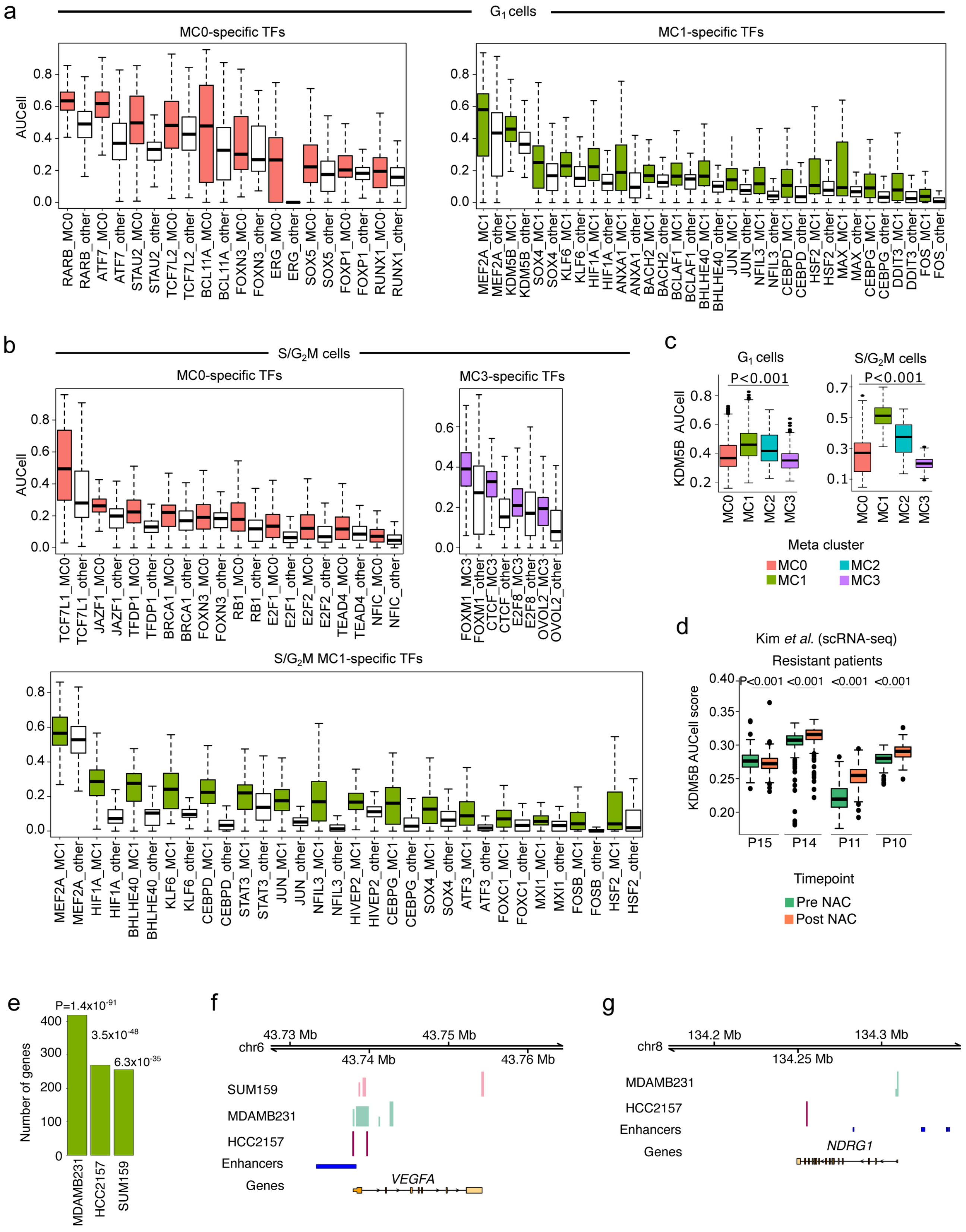

Fig.S6. Transcriptional factors (TFs) drive transcriptional identity of MCs. a-b. Box plots of AUCell score obtained from SCENIC show MC-specific TF/TF regulators of G_1 (a) and S/G_2M epithelial cells (b). **c**. Box plots of AUCell score of *KDM5B* in G_1 (left) and S/G_2M (right) epithelial cells show higher regulation of KDM5B in hypoxia MCs (G_1-MC1 and S/G_2M-MC1; Kruskal–Wallis test). **d**. Box plots of AUCell score of KDM5B transcription factor (TF) regulator in resistant and sensitive patients pre and post NAC in an independent single cell dataset show significant enrichment of KDM5B regulation post chemotherapy in most of resistant patients but not in sensitive patients (Wilcoxon test). **e**. Bar plot of the number of the promoter regions of the top 500 marker genes of G_1-MC1 bound by KDM5B. P-values represent enrichment (hypergeometric test). **f-g**. Representative examples of peak scores of the chromosomal regions of G_1-MC1 marker genes *VEGFA* (f) and *NDRG1* (g) across different TNBC cell lines subjected to KDM5B ChIP-seq.

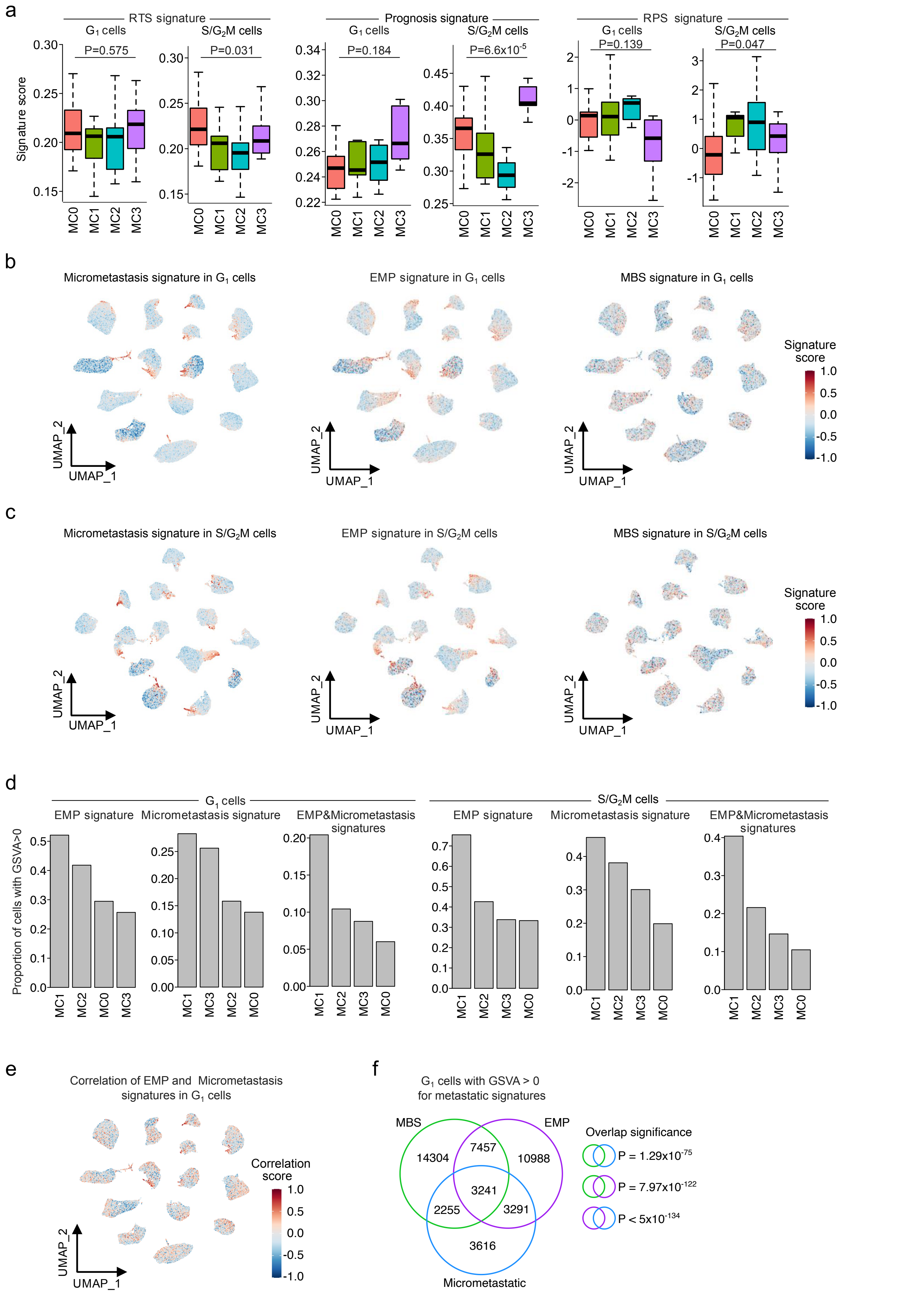

Fig.S7. Gene signatures characterised chromosomal instability and DNA damage-dominant cell states and cells with metastasis potential. a. Signature score of Residual signature (RTS), prognosis signature, and Recombination proficiency

score (RPS) on G_1 and S/G_2M epithelial cells (Kruskal-Wallis test). **b-c.** UMAP visualisation of micrometastasis (left), Epithelial Mesenchymal Plasticity (EMP) (middle), and Metastatic Burden Signature (MBS) (left) GSVA signature scores in G_1 (b) and S/G_2M cells (c). A small population of cells show high GSVA scores of micrometastasis and EMP signatures in G_1 and S/G_2M epithelial cells. No cluster of cells show MBS specificity in G_1 and S/G_2M epithelial cells. The negative GSVA scores were scaled to [-1,0) and the positive GSVA scores were scaled to (0,1] within each sample for visualisation. **d.** Bar plots of fraction of cells expressing (positive GSVA) EMP signature, micrometastasis signature, and both EMP & micrometastasis signatures in G_1 and S/G_2M epithelial cells MCs, showing that hypoxia MCs (G_1-MC1 and S/G_2M-MC1) contain larger fraction of cells with high metastasis potential. **e.** UMAP visualisation of cell-wise correlation of micrometastasis and EMP signature scores GSVA signature scores in G_1 cells. The correlation scores were scaled to [-1,1] for visualisation. **f.** Venn plot showing the number of G_1 cells expressing EMP, micrometastasis, and EMP signatures. P values show the overlap significance between cells expressing each two signatures based on hypergeometric test.

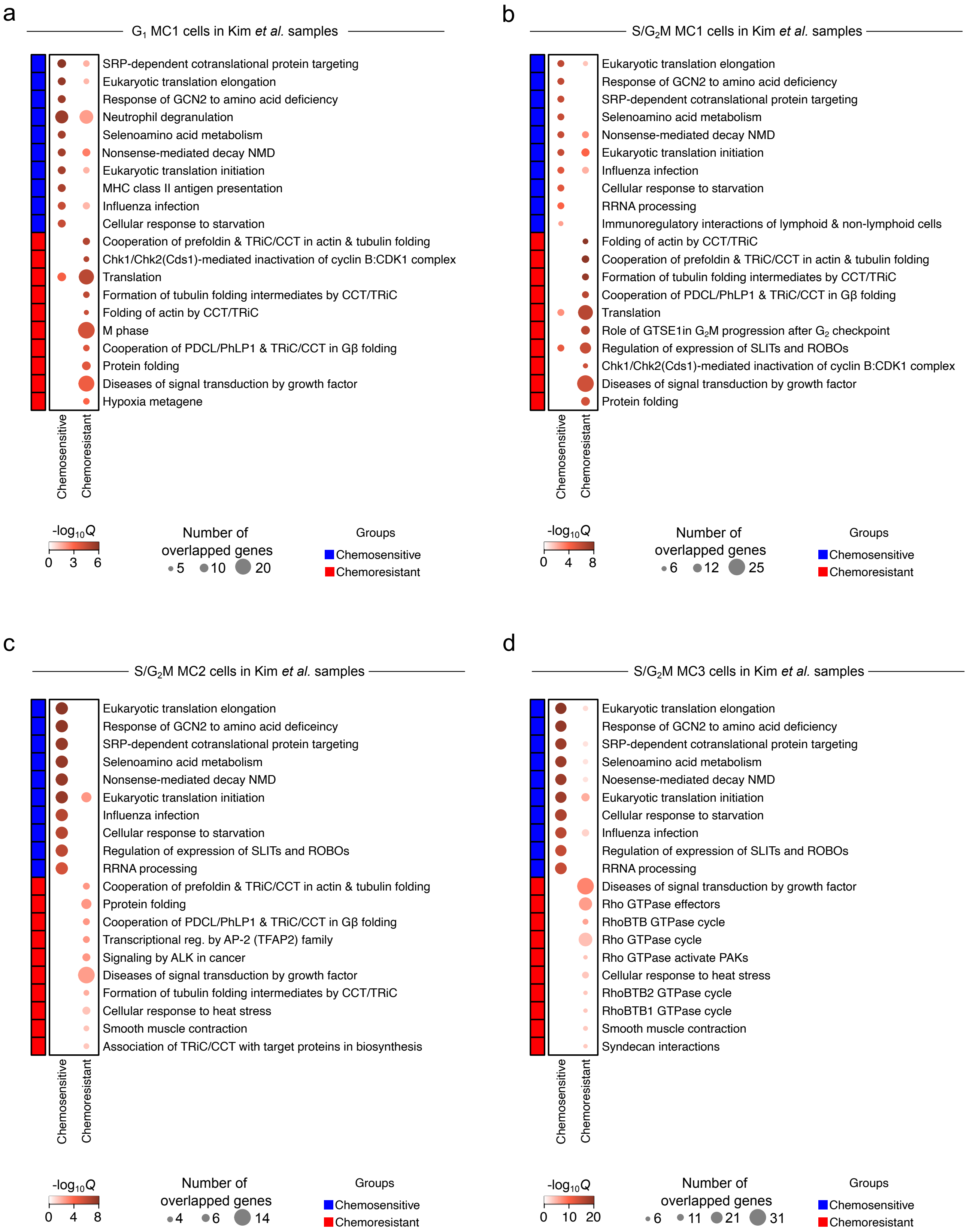
a
G1 MC1 cells in Kim et al. samples
SRP-dependent cotranslational protein targeting
Eukaryotic translation elongation
Response of GCN2 to amino acid deficiency
Neutrophil degranulation
Selenoamino acid metabolism
Nonsense-mediated decay NMD
Eukaryotic translation initiation
MHC class II antigen presentation
Influenza infection
Cellular response to starvation
Cooperation of prefoldin & TRiC/CCT in actin & tubulin folding
Chk1/Chk2(Cds1)-mediated inactivation of cyclin B:CDK1 complex
Translation
Formation of tubulin folding intermediates by CCT/TRiC
Folding of actin by CCT/TRiC
M phase
Cooperation of PDCL/PhLP1 & TRiC/CCT in Gβ folding
Protein folding
Diseases of signal transduction by growth factor
Hypoxia metagene
Chemosensitive
Chemoresistant
-log10Q
0 3 6
Number of overlapped genes
5 10 20
Groups
Chemosensitive
Chemoresistant
b
S/G2M MC1 cells in Kim et al. samples
Eukaryotic translation elongation
Response of GCN2 to amino acid deficiency
SRP-dependent cotranslational protein targeting
Selenoamino acid metabolism
Nonsense-mediated decay NMD
Eukaryotic translation initiation
Influenza infection
Cellular response to starvation
RRNA processing
Immunoregulatory interactions of lymphoid & non-lymphoid cells
Folding of actin by CCT/TRiC
Cooperation of prefoldin & TRiC/CCT in actin & tubulin folding
Formation of tubulin folding intermediates by CCT/TRiC
Cooperation of PDCL/PhLP1 & TRiC/CCT in Gβ folding
Translation
Role of GTSE1in G2M progression after G2 checkpoint
Regulation of expression of SLITs and ROBOs
Chk1/Chk2(Cds1)-mediated inactivation of cyclin B:CDK1 complex
Diseases of signal transduction by growth factor
Protein folding
Chemosensitive
Chemoresistant
-log10Q
0 4 8
Number of overlapped genes
6 12 25
Groups
Chemosensitive
Chemoresistant
c
S/G2M MC2 cells in Kim et al. samples
Eukaryotic translation elongation
Response of GCN2 to amino acid deficiency
SRP-dependent cotranslational protein targeting
Selenoamino acid metabolism
Nonsense-mediated decay NMD
Eukaryotic translation initiation
Influenza infection
Cellular response to starvation
Regulation of expression of SLITs and ROBOs
RRNA processing
Cooperation of prefoldin & TRiC/CCT in actin & tubulin folding
Pprotein folding
Cooperation of PDCL/PhLP1 & TRiC/CCT in Gβ folding
Transcriptional reg. by AP-2 (TFAP2) family
Signaling by ALK in cancer
Diseases of signal transduction by growth factor
Formation of tubulin folding intermediates by CCT/TRiC
Cellular response to heat stress
Smooth muscle contraction
Association of TRiC/CCT with target proteins in biosynthesis
Chemosensitive
Chemoresistant
-log10Q
0 4 8
Number of overlapped genes
4 6 14
Groups
Chemosensitive
Chemoresistant
d
S/G2M MC3 cells in Kim et al. samples
Eukaryotic translation elongation
Response of GCN2 to amino acid deficiency
SRP-dependent cotranslational protein targeting
Selenoamino acid metabolism
Noesense-mediated decay NMD
Eukaryotic translation initiation
Cellular response to starvation
Influenza infection
Regulation of expression of SLITs and ROBOs
RRNA processing
Diseases of signal transduction by growth factor
Rho GTPase effectors
RhoBTB GTPase cycle
Rho GTPase cycle
Rho GTPase activate PAKs
Cellular response to heat stress
RhoBTB2 GTPase cycle
RhoBTB1 GTPase cycle
Smooth muscle contraction
Syndecan interactions
Chemosensitive
Chemoresistant
-log10Q
0 10 20
Number of overlapped genes
6 11 21 31
Groups
Chemosensitive
Chemoresistant

Fig.S8. Cell states characterised from residual disease harboured differential pathway activation between sensitive and resistant primary naïve tumours. a-d. Pathway enrichment for the top 10 most significant pathways in G_1-MC1-positive (a), S/G_2M-MC1-positive (b), S/G_2M-MC2-positive (c), and S/G_2M-MC3-positive (c) cancer cells in treatment-naïve chemosensitive vs chemoresistant patients in a public single cell dataset[2] using the Reactome database in MSigDB. The colour of dot represents $-\log_{10}$(FDR-adjusted P) and the size of dot represents the number of genes intersected by top 100 marker genes and pathway gene sets.

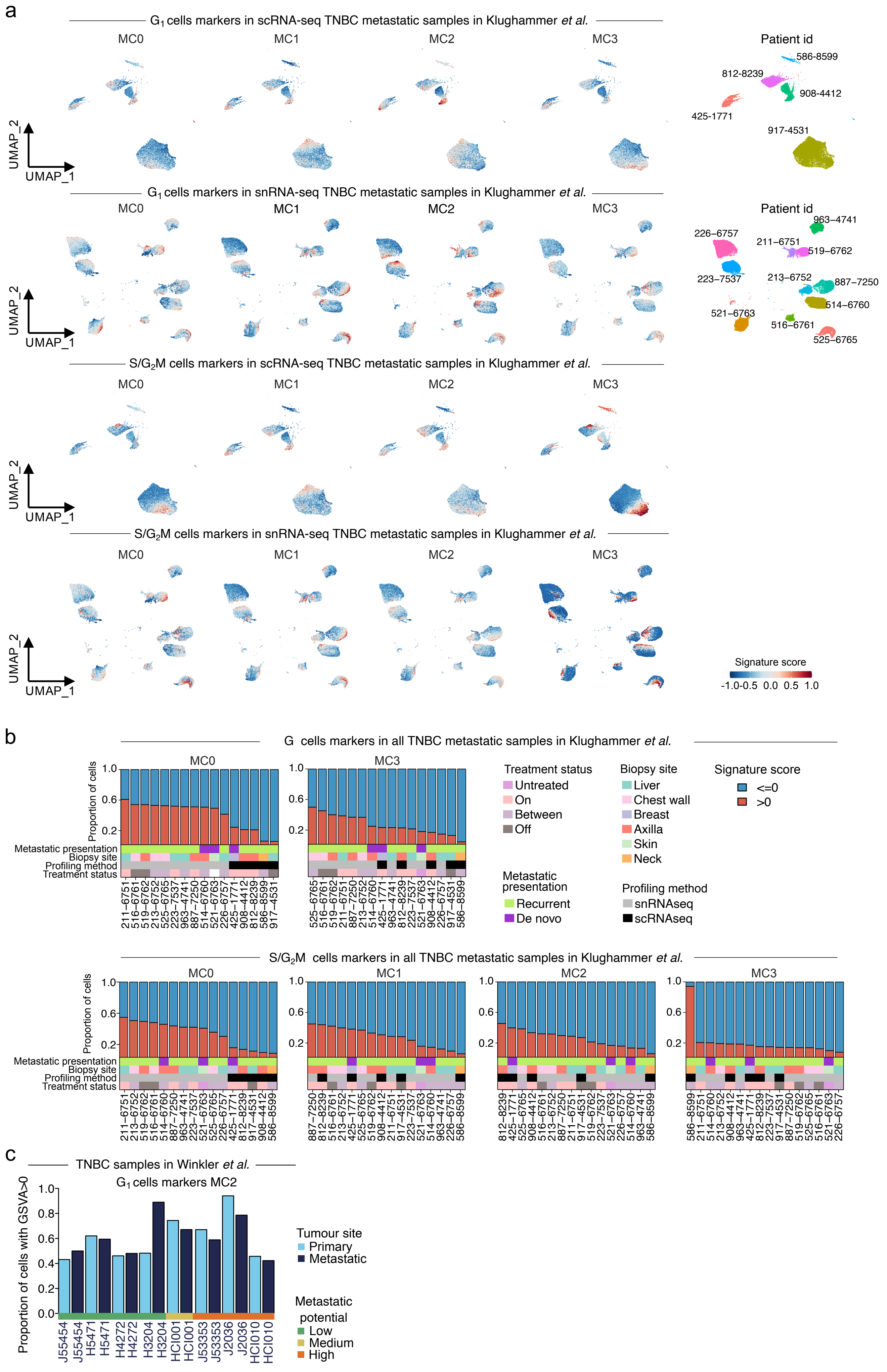
a
G1 cells markers in scRNA-seq TNBC metastatic samples in Klughammer et al.
MC0
MC1
MC2
MC3
Patient id
586-8599
812-8239
908-4412
425-1771
917-4531
UMAP_2
UMAP_1
G1 cells markers in snRNA-seq TNBC metastatic samples in Klughammer et al.
963–4741
226–6757
211–6751
519–6762
223–7537
213–6752
887–7250
514–6760
521–6763
516–6761
525–6765
S/G2M cells markers in scRNA-seq TNBC metastatic samples in Klughammer et al.
S/G2M cells markers in snRNA-seq TNBC metastatic samples in Klughammer et al.
Signature score
-1.0 -0.5 0.0 0.5 1.0
b
G1 cells markers in all TNBC metastatic samples in Klughammer et al.
Proportion of cells
Metastatic presentation
Biopsy site
Profiling method
Treatment status
Treatment status
Untreated
On
Between
Off
Biopsy site
Liver
Chest wall
Breast
Axilla
Skin
Neck
Signature score
<=0
>0
Metastatic presentation
Recurrent
De novo
Profiling method
snRNAseq
scRNAseq
S/G2M cells markers in all TNBC metastatic samples in Klughammer et al.
c
TNBC samples in Winkler et al.
G1 cells markers MC2
Proportion of cells with GSVA>0
J55454
J55454
H5471
H5471
H4272
H4272
H3204
H3204
HCI001
HCI001
J53353
J53353
J2036
J2036
HCI010
HCI010
Tumour site
Primary
Metastatic
Metastatic potential
Low
Medium
High

Fig.S9. Cell states characterised from residual disease are maintained in metastatic TNBC tumours. Cell states characterised from residual disease are maintained in metastatic TNBC tumours in a public single cell dataset[29]. **a.** Signature scores (GSVA score on top 100 marker genes) of G_1 and S/G_2M epithelial cells MCs highlighted distinct subpopulations in metastatic TNBC tumour cells on UMAP. The negative GSVA scores were scaled to [-1,0) and the positive GSVA scores were scaled to (0,1] within each sample for visualisation. **b.** Bar plots show fraction of cells expressing MC signatures (GSVA > 0) of G_1 and S/G_2M epithelial cells from residual disease in metastatic TNBC patients. **c.** Bar plot of proportion of cells expressing G_1-MC1 signature (GSVA > 0) in paired primary and metastatic PDX basal-like TNBC models from Winkler *et al.*[3] showing persister-like MC1 is maintained or enriched in metastatic disease.

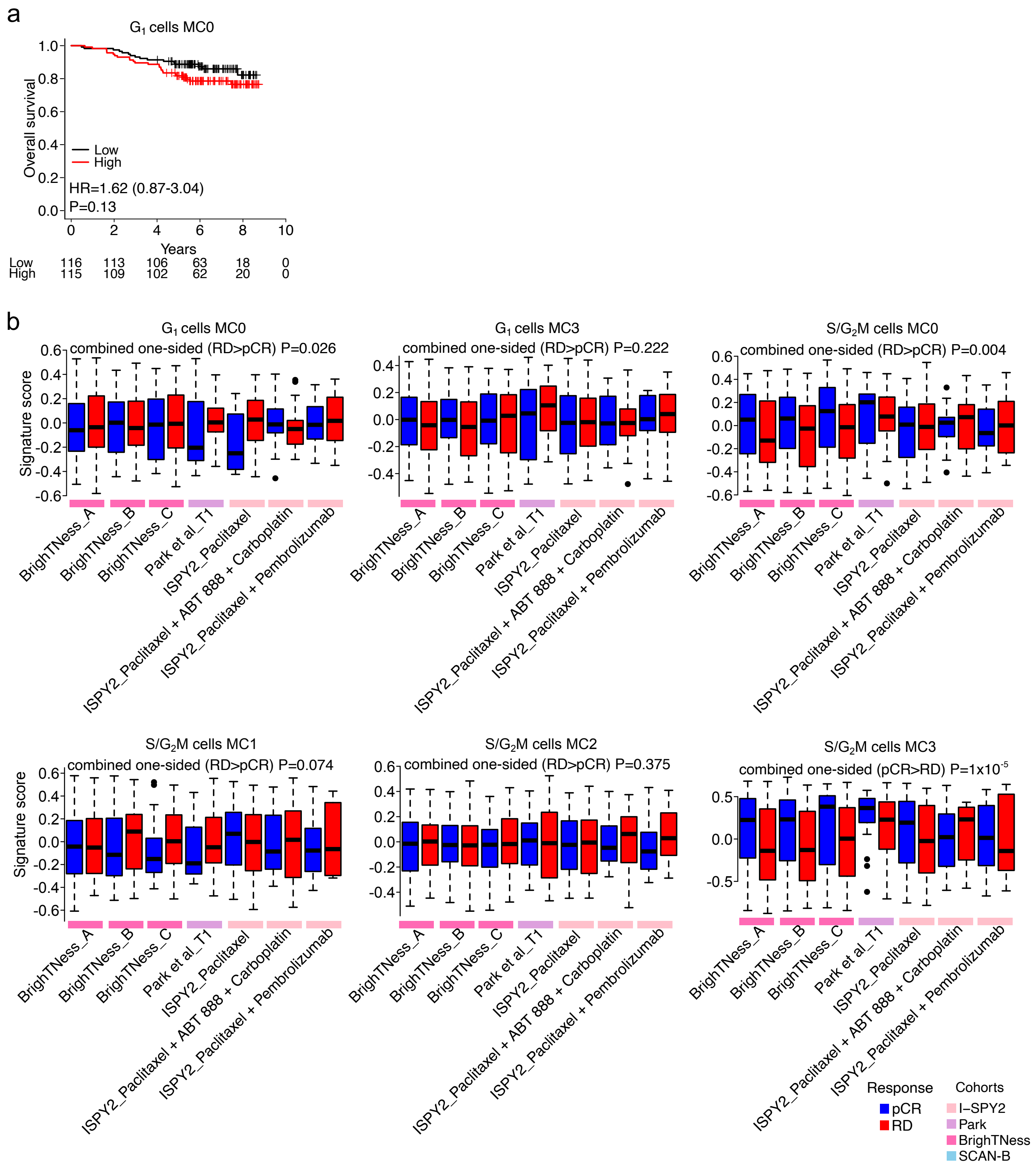

Fig.S10. Subclonal markers in NAC residual disease predict pathological complete response in primary untreated TNBC as gene signature. a. Kaplan-Meier plot of G_1 cells MC0 (GSVA score of top 100 marker genes) in SCAN-B chemotherapy TNBC patients show association with poor and good prognosis respectively, low: <= 50% quantile, high: > 50% quantile of GSVA score. **b.** Box plot of signature scores (GSVA score of top 100 marker genes) of chemo naïve residual disease (RD) and pathological complete response (pCR) patients from seven arms with standard treatments (chemotherapy and/or immunotherapy) on G_1 and S/G_2M cells MCs (combined P-value of weighted sum of z method on one-side Wilcoxon test).

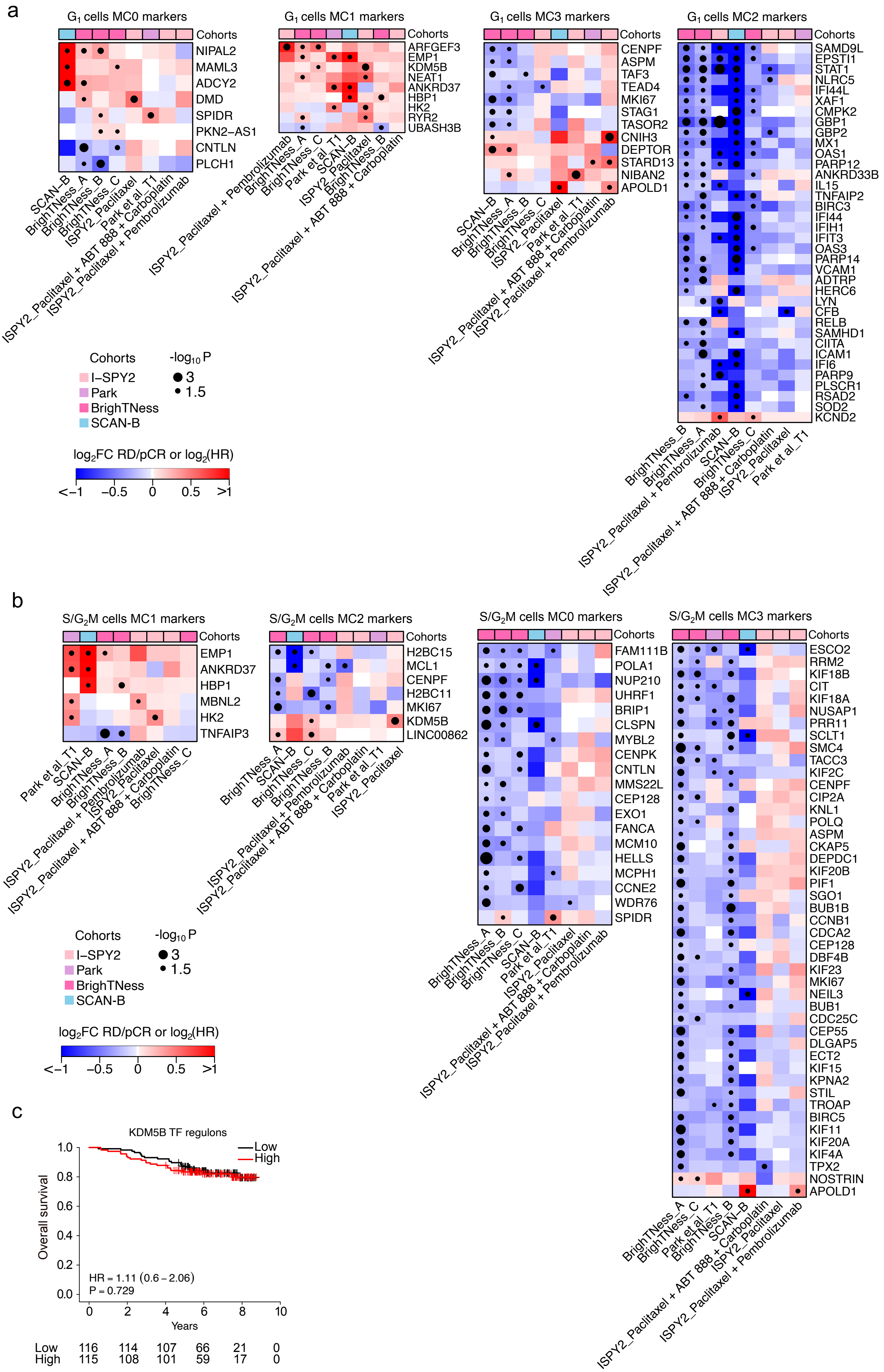
a
G1 cells MC0 markers
Cohorts
NIPAL2
MAML3
ADCY2
DMD
SPIDR
PKN2-AS1
CNTLN
PLCH1
SCAN-B
BrighTNess_A
BrighTNess_B
BrighTNess_C
ISPY2_Paclitaxel
Park et al_T1
ISPY2_Paclitaxel + ABT 888 + Carboplatin
ISPY2_Paclitaxel + Pembrolizumab
G1 cells MC1 markers
Cohorts
ARFGEF3
EMP1
KDM5B
NEAT1
ANKRD37
HBP1
HK2
RYR2
UBASH3B
ISPY2_Paclitaxel + Pembrolizumab
BrighTNess_A
BrighTNess_C
Park et al_T1
SCAN-B
ISPY2_Paclitaxel
BrighTNess_B
ISPY2_Paclitaxel + ABT 888 + Carboplatin
G1 cells MC3 markers
Cohorts
CENPF
ASPM
TAF3
TEAD4
MKI67
STAG1
TASOR2
CNIH3
DEPTOR
STARD13
NIBAN2
APOLD1
SCAN-B
BrighTNess_A
BrighTNess_B
BrighTNess_C
ISPY2_Paclitaxel
Park et al_T1
ISPY2_Paclitaxel + ABT 888 + Carboplatin
ISPY2_Paclitaxel + Pembrolizumab
G1 cells MC2 markers
Cohorts
SAMD9L
EPSTI1
STAT1
NLRC5
IFI44L
XAF1
CMPK2
GBP1
GBP2
MX1
OAS1
PARP12
ANKRD33B
IL15
TNFAIP2
BIRC3
IFI44
IFIH1
IFIT3
OAS3
PARP14
VCAM1
ADTRP
HERC6
LYN
CFB
RELB
SAMHD1
CIITA
ICAM1
IFI6
PARP9
PLSCR1
RSAD2
SOD2
KCND2
BrighTNess_B
BrighTNess_A
ISPY2_Paclitaxel + Pembrolizumab
SCAN-B
BrighTNess_C
ISPY2_Paclitaxel + ABT 888 + Carboplatin
ISPY2_Paclitaxel
Park et al_T1
Cohorts
I–SPY2
Park
BrighTNess
SCAN-B
-log10 P
3
1.5
log2FC RD/pCR or log2(HR)
<–1
–0.5
0
0.5
>1
b
S/G2M cells MC1 markers
Cohorts
EMP1
ANKRD37
HBP1
MBNL2
HK2
TNFAIP3
Park et al_T1
SCAN-B
BrighTNess_A
BrighTNess_B
ISPY2_Paclitaxel + Pembrolizumab
ISPY2_Paclitaxel
ISPY2_Paclitaxel + ABT 888 + Carboplatin
BrighTNess_C
S/G2M cells MC2 markers
Cohorts
H2BC15
MCL1
CENPF
H2BC11
MKI67
KDM5B
LINC00862
BrighTNess_A
SCAN-B
BrighTNess_C
BrighTNess_B
ISPY2_Paclitaxel + Pembrolizumab
ISPY2_Paclitaxel + ABT 888 + Carboplatin
Park et al_T1
ISPY2_Paclitaxel
S/G2M cells MC0 markers
Cohorts
FAM111B
POLA1
NUP210
UHRF1
BRIP1
CLSPN
MYBL2
CENPK
CNTLN
MMS22L
CEP128
EXO1
FANCA
MCM10
HELLS
MCPH1
CCNE2
WDR76
SPIDR
BrighTNess_A
BrighTNess_B
BrighTNess_C
SCAN-B
Park et al_T1
ISPY2_Paclitaxel
ISPY2_Paclitaxel + ABT 888 + Carboplatin
ISPY2_Paclitaxel + Pembrolizumab
S/G2M cells MC3 markers
Cohorts
ESCO2
RRM2
KIF18B
CIT
KIF18A
NUSAP1
PRR11
SCLT1
SMC4
TACC3
KIF2C
CENPF
CIP2A
KNL1
POLQ
ASPM
CKAP5
DEPDC1
KIF20B
PIF1
SGO1
BUB1B
CCNB1
CDCA2
CEP128
DBF4B
KIF23
MKI67
NEIL3
BUB1
CDC25C
CEP55
DLGAP5
ECT2
KIF15
KPNA2
STIL
TROAP
BIRC5
KIF11
KIF20A
KIF4A
TPX2
NOSTRIN
APOLD1
BrighTNess_A
BrighTNess_C
Park et al_T1
BrighTNess_B
SCAN-B
ISPY2_Paclitaxel + ABT 888 + Carboplatin
ISPY2_Paclitaxel
ISPY2_Paclitaxel + Pembrolizumab
Cohorts
I–SPY2
Park
BrighTNess
SCAN-B
-log10 P
3
1.5
log2FC RD/pCR or log2(HR)
<–1
–0.5
0
0.5
>1
c
KDM5B TF regulons
Low
High
Overall survival
1.0
0.8
0.6
0.4
0.2
0.0
HR = 1.11 (0.6 – 2.06)
P = 0.729
0
2
4
6
8
10
Years
Low 116 114 107 66 21 0
High 115 108 101 59 17 0

Fig.S11. Marker genes of subclonal alternations in NAC residual disease predict pathological complete response in primary untreated TNBC. a-b. Heatmap of log_2(HR) or log_2 fold change (log_2FC) (RD divided by pCR) on top 100 marker genes which are significant in at least two arms or cohorts and have same directions of log_2(HR) and log_2FC on significant genes from G_1 cells MCs (a) and S/G_2M cells MCs (b). The dot represents -log_{10}(*P*). **c**. Kaplan-Meier plot of GSVA score of KDM5B regulons in SCAN-B chemotherapy TNBC patients, low: <= 50% quantile, high: > 50% quantile of GSVA score**.**

1. Staaf, J., Häkkinen, J., Hegardt, C., Saal, L. H., Kimbung, S., Hedenfalk, I., Lien, T., Sørlie, T., Naume, B., Russnes, H., Marcone, R., Ayyanan, A., Brisken, C., Malterling, R. R., Asking, B., Olofsson, H., Lindman, H., Bendahl, P. O., Ehinger, A., . . . Vallon-Christersson, J. (2022). RNA Sequencing-based single sample predictors of molecular subtype and risk of recurrence for clinical assessment of early-stage breast cancer. Mendeley Data V1. 10.17632/yzxtxn4nmd.1.
2. Kim, C., Gao, R., Sei, E., Brandt, R., Hartman, J., Hatschek, T., Crosetto, N., Foukakis, T., and Navin, N.E. (2018). Chemoresistance evolution in triple-negative breast cancer delineated by single-cell sequencing. Cell *173*, 879-893. e813.
3. Winkler, J., Tan, W., Diadhiou, C.M., McGinnis, C.S., Abbasi, A., Hasnain, S., Durney, S., Atamaniuc, E., Superville, D., Awni, L., et al. (2024). Single-cell analysis of breast cancer metastasis reveals epithelial-mesenchymal plasticity signatures associated with poor outcomes. J Clin Invest *134*. 10.1172/jci164227.
